# Prediction of hypertension and restenosis under guideline-directed management in aortic coarctation: development and validation of machine-learning models

**DOI:** 10.1016/j.eclinm.2026.104041

**Published:** 2026-06-26

**Authors:** Lea Fierley, Jakob Versnjak, Grischa Gabel, Peter Kramer, Leonid Goubergrits, Felix Berger, Grégoire Montavon, Titus Kuehne, Marcus Kelm

**Affiliations:** a Institute of Computer-Assisted Cardiovascular Medicine, Deutsches Herzzentrum der Charité Augustenburger Platz 1, 13353 Berlin, Germany; b Department of Congenital Heart Disease – Pediatric Cardiology, Deutsches Herzzentrum der Charité, Augustenburger Platz 1, 13353 Berlin, Germany; c DZHK (German Centre for Cardiovascular Research), Partner Site Berlin, Germany; d Charité - Universitätsmedizin Berlin, Corporate Member of Freie Universität Berlin and Humboldt-Universität zu Berlin, Charitéplatz 1, 10117 Berlin, Germany; e BIFOLD – Berlin Institute for the Foundations of Learning and Data, Berlin, Germany

**Keywords:** Aortic coarctation, Arterial hypertension, Machine learning, Decision support, Congenital heart disease, Explainable artificial intelligence

## Abstract

**Background:**

Aortic coarctation (CoA) is a major cause of arterial hypertension in young individuals, with recurrence occurring in up to one-third of patients throughout life despite guideline-directed management.

**Methods:**

We conducted a development and validation study at a single centre in Berlin, Germany, utilising routinely collected electronic health records, cardiovascular magnetic resonance (CMR), and mid-/long-term follow-up data from 218 visits (160 individuals with CoA receiving standard of care, guideline-based management) collected between January 2014 and April 2022. Machine learning (ML) models (CatBoost, XGBoost, random forest, support vector classifiers, neural networks, logistic regression, and K-nearest neighbours) were developed to predict three endpoints: re-coarctation requiring intervention (CoA-I), aortic surgery (CoA-S) as a subset of CoA-I, and persistent arterial hypertension. The dataset was divided by random stratified split into a training set (n = 159; for model development with five-fold cross-validation), and a hold-out test set (n = 59; for out-of-sample validation). Stratification was based on sex, age, and CoA-I status. We included a final set of 38 clinically relevant features, encompassing baseline characteristics, medication intake, echocardiography, CMR, electrocardiography (ECG), and treatment decisions. ClinicalTrials.gov Identifier: NCT02591940.

**Findings:**

Tree-based and support vector classifier models performed best after Bayesian hyperparameter optimisation, yielding high performance in a stratified validation cohort: area under the receiver operating characteristic curve (ROC AUC) 0.90 ± 0.01 for CoA-I, 0.90 ± 0.01 for CoA-S, and 0.84 ± 0.01 for hypertension. Shapley Additive exPlanations (SHAP) highlighted peak Doppler gradient, time since index visit, and ventricular size indices as key predictors for CoA-I. In inverse-probability-weighted analyses, antihypertensive medication was associated with a lower CoA-I probability (−17.3%; 95% confidence interval [CI], −28.2 to −6.4; p = 0.002), with concordant propensity-score-matched findings. An open-access research interface (https://icm.dhzc.charite.de/calc_coa) incorporates treatment thresholds and personalised risk estimates from an updatable ML framework.

**Interpretation:**

These findings suggest that patient-specific multimodal ML-based risk estimates may complement guideline-based care by identifying patients at increased risk of CoA-I or persistent hypertension, with the potential to support more tailored follow-up and reduce lifetime exposure to brachiocephalic hypertension. In adjusted cohort-level analyses, antihypertensive medication was associated with a lower probability of CoA-I.

**Funding:**

This study was supported by the European Commission's Seventh Framework Programme (FP7, project ID 611232). M.K. acknowledges support within the Charité Digital Clinician Scientist Programme funded by DFG. M.K. and T.K. have received funding within the CHAIN project (Project ID: 101314833), supported by the European Union's EU4Health Programme. T.K. and M.K. acknowledge support within the Collaborative Research Centre SFB 1470, funded by the Deutsche Forschungsgemeinschaft (DFG, German Research Foundation), project ID 437531118. M.K. has received funding from the Bundesministerium für Forschung, Technologie und Raumfahrt (BMFTR, Federal Ministry of Research, Technology and Space), VADYS-ME, grant number 01EJ2406A.

**Research in context**

**Evidence before this study**

We searched PubMed from database inception (or start date) to May 11, 2026, for papers published in English (or with no language restrictions), using the terms “coarctation of the aorta”, “recoarctation”, “machine learning”, “artificial intelligence”, “prediction model”, and “risk score”. Our search yielded 998 results. Advances in machine learning have improved prognostication across several cardiovascular conditions, but no widely accepted and validated risk model currently exists for congenital heart disease. In aortic coarctation, persistent arterial hypertension and residual haemodynamic burden remain major drivers of long-term morbidity and later reintervention despite guideline-directed management.

**Added value of this study**

Using routinely collected electronic health records, echocardiography, cardiovascular magnetic resonance, and follow-up data from 218 visits in 160 individuals with aortic coarctation, we developed and validated machine-learning classifiers for re-coarctation requiring intervention, persistent arterial hypertension, and aortic surgery as a subset of re-coarctation. The models generate patient-specific probabilities, identify key predictors using Shapley Additive exPlanations, and are translated into an open-access research interface that combines guideline thresholds with personalised risk estimates for routine visits in aortic coarctation. In population-based analyses, inverse-probability weighting with concordant propensity-score-matched findings showed that antihypertensive medication was associated with a lower probability of re-coarctation requiring intervention.

**Implications of all the available evidence**

The available evidence suggests that routine clinical and imaging data can support risk stratification for routine visits in aortic coarctation. Our findings indicate that patient-specific multimodal machine-learning risk estimates may complement guideline-based care by identifying patients at increased risk of re-coarctation requiring intervention or persistent arterial hypertension. In addition, population-based adjusted analyses suggest that antihypertensive medication may be associated with a lower probability of future re-coarctation requiring intervention. The added clinical value of this approach requires prospective multicentre evaluation and should not replace clinical judgement.

## Introduction

Aortic coarctation (CoA) represents a main cause of arterial hypertension in children and young adults, often resulting in increased cardiovascular morbidity and mortality.^1^, ^2^, ^3^, ^4^, ^5^ Despite advances in surgical, catheter-based, and medical treatment strategies, hypertension - often silent and not limited to cases with re-coarctation - remains a driver of stroke, coronary artery disease, and heart failure.^3^^,^^5^ It persists in up to one-third of patients after repair.^4^ Consequently, lifelong surveillance is required, yet only 20% of patients remain normotensive and free from complications and reintervention on long-term follow-up.^3^

Timely identification of at-risk patients is therefore essential. Machine learning (ML) approaches have improved prognostication across several cardiovascular conditions,^6^, ^7^, ^8^, ^9^, ^10^ and traditional risk scores such as the Framingham score and EuroSCORE have been available for decades.^11^^,^^12^ However, no such widely accepted and validated risk model currently exists for congenital heart disease (CHD). Although CoA is particularly relevant to the development of arterial hypertension, CHD remains relatively rare in the general population, and this low prevalence restricts the development of disease-specific risk scores. In this context, the use of routinely collected healthcare data in combination with ML techniques offers a promising approach to uncovering important predictive features for health outcomes. While prospective observational studies are often more limited in duration and sample size, hospital-based data systems enable continuous case identification and integration into ML applications.

We therefore sought to develop and validate ML models trained on electronic health records, echocardiographic and cardiovascular magnetic resonance (CMR) data, to predict two main clinical outcomes: persistent arterial hypertension and the composite outcome of re-coarctation requiring intervention (CoA-I). Re-coarctation necessitating aortic surgery (CoA-S) was analysed separately as a secondary surgical outcome. In addition, we aimed to model treatment selection.

## Methods

### Study design and population

This prospective observational study was conducted at Deutsches Herzzentrum der Charité/Berlin in accordance with a pre-registered protocol (ClinicalTrials.gov, NCT02591940). Individuals meeting the inclusion criteria, namely a confirmed diagnosis of CoA and an indication for imaging-based diagnostic evaluation,^13^^,^^14^ underwent the initial diagnostic work-up and were enrolled in the analysis. Additional mid-/long-term outcomes were subsequently incorporated into the final analyses.

Patients were seen for echocardiography in dedicated outpatient clinics and referred for CMR in cases of limited acoustic window, elevated pressure gradients on echocardiography, or as part of routine post-interventional follow-up. Both the outpatient clinic and the imaging facility use a unified institutional reporting system (Centricity™ Cardio Workflow V.7.0, GE HealthCare, Chicago, IL, USA). Parametric imaging data and structured clinical findings from the reporting system, as well as longitudinal follow-up records maintained in a REDCap research database, were queried for analysis.^15^ All institutional follow-up data were included, although an absolute follow-up period was calculated specifically for visits with an observation interval of at least one month following the index visit. Patients who did not attend institutional or known external follow-up remained labelled as reintervention-free unless data indicated otherwise. The resulting dataset enabled the consolidation of clinical variables, imaging parameters, and follow-up data, allowing for linkage with predefined study outcomes.

While the study population comprised patients diagnosed with CoA between January 2014 and April 2021, each follow-up dataset (collected until April 2022) was considered a distinct case, defined by unique clinical, imaging and outcome characteristics. Follow-up visits occurring less than six months after the previous intervention were excluded to mitigate potential carry-over effects from the prior treatment, thereby ensuring that each assessment accurately reflected the patient's current clinical status. Following these exclusions, we performed a visit-level random stratified split of the dataset into (i) a development set, utilised for model training and optimisation and (ii) a separate hold-out test set, strictly reserved for final out-of-sample validation. Stratification was based on sex, age, and CoA-I status to ensure preservation of the marginal distributions of these variables within each subset (Table 1).

**Table 1:** Baseline clinical characteristics of the study population.

	Total (n = 218)	Development (n = 159)	Validation (n = 59)	p-value	CoA-I (n = 57)	Non-CoA-I (n = 161)	p-value
**Characteristics**							
Age (months)	218 (153.25, 337.5)	218 (153.5, 355)	214 (153.5, 298.5)	0.25	179 (115, 252)	228 (16, 354)	<0.01
Age (years)	18 (13, 28)	18.00 (13, 30)	18 (12.5, 24.5)	0.24	15 (10, 21)	19 (13, 30)	<0.01
Male (n; %)	141 (64.7%)	101 (63.5%)	40 (67.8%)	0.67	40 (70.2%)	101 (62.7%)	0.40
Weight (kg)	65 (45, 78)	66 (48, 79.3)	65 (39.55, 73.5)	0.30	57 (31, 68)	68 (49.5, 80)	<0.01
Height (cm)	169 (152, 178)	168.5 (153, 177.75)	169 (150, 179.5)	0.85	158 (140, 173)	171 (156.75, 180)	<0.01
BMI (kg/m^2^)	22 (18.1, 25.5)	22.4 (18.4, 25.6)	20.9 (17.75, 24.8)	0.087	20.4 (17.3, 23.5)	22.55 (18.98, 25.82)	<0.01
BSA (m^2^)	1.73 (1.37, 1.96)	1.73 (1.43, 1.97)	1.72 (1.32, 1.92)	0.41	1.58 (1.1, 1.81)	1.82 (1.46, 1.98)	<0.01
AVD (n; %)	119 (54.6%)	82 (51.6%)	37 (62.7%)	0.19	34 (59.6%)	85 (52.8%)	0.46
AVD with isolated aortic stenosis (n; %)	16 (7.3%)	10 (6.3%)	6 (10.2%)	0.38	4 (7.0%)	12 (7.5%)	1.00
AVD with isolated aortic regurgitation (n; %)	40 (18.3%)	28 (17.6%)	12 (20.3%)	0.79	18 (31.6%)	22 (13.7%)	<0.01
AVD with combined aortic stenosis and regurgitation (n; %)	14 (6.4%)	9 (5.7%)	5 (8.5%)	0.53	1 (1.8%)	13 (8.1%)	0.12
Bicuspid aortic valve (n; %)	107 (49.1%)	74 (46.5%)	33 (55.9%)	0.28	26 (45.6%)	81 (50.3%)	0.65
Mitral valve disease (n; %)	30 (13.8%)	21 (13.2%)	9 (15.3%)	0.87	14 (24.6%)	16 (9.9%)	0.011
Ascending aortic aneurysm (n; %)	30 (13.8%)	21 (13.2%)	9 (15.3%)	0.87	5 (8.8%)	25 (15.5%)	0.29
Arterial hypertension (n; %)	120 (55.0%)	85 (53.5%)	35 (59.3%)	0.54	32 (56.1%)	88 (54.7%)	0.97
Resting heart rate (bpm)	71 (62.5, 86)	73 (62, 85)	70 (65, 89.25)	0.88	72.5 (65, 86.25)	71 (62, 85)	0.53
Blood pressure gradient (mmHg)	2.5 (−7.62, 13.75)	1.5 (−8.75, 13)	5.5 (−3.5, 15)	0.19	5.5 (−5, 18)	2.50 (−8, 13)	0.51
Systolic blood pressure percentile	96 (84, 99)	96 (84, 99)	96 (86, 99)	0.44	97 (92.25, 99)	95.5 (82, 99)	0.072
Diastolic blood pressure percentile	50 (50, 75.5)	50.00 (50, 75)	50 (50, 77.5)	0.64	50 (41.5, 81.5)	50 (50, 75)	0.61
ECG QTC (ms)	430 (408, 447)	428 (408, 448)	430 (409, 445)	0.53	430 (411, 445)	426 (408, 449.5)	0.64
ECG QRS (ms)	100 (86, 108)	100 (86, 108.5)	94 (86, 106)	0.69	90 (80, 100)	100 (88.5, 110)	0.047
**Previous intervention**							
Valve surgery (n; %)	17 (7.8%)	13 (8.2%)	4 (6.8%)	1.00	4 (7.0%)	13 (8.1%)	1.00
Any aortic intervention (n; %)	165 (75.7%)	121 (76.1%)	44 (74.6%)	0.96	41 (71.9%)	124 (77.0%)	0.56
Balloon angioplasty (n; %)	111 (50.9%)	85 (53.5%)	26 (44.1%)	0.28	32 (56.1%)	79 (49.1%)	0.45
Stenting (n; %)	67 (30.7%)	51 (32.1%)	16 (27.1%)	0.59	16 (28.1%)	51 (31.7%)	0.73
Surgical reconstruction (n; %)	100 (45.9%)	72 (45.3%)	28 (47.5%)	0.89	25 (43.9%)	75 (46.6%)	0.84
**Medication**							
ACE inhibitors (n; %)	60 (27.5%)	47 (29.6%)	13 (22.0%)	0.35	16 (28.1%)	44 (27.3%)	1.00
Angiotensin receptor blockers (n; %)	15 (6.9%)	14 (8.8%)	1 (1.7%)	0.075	3 (5.3%)	12 (7.5%)	0.76
Beta blockers (n; %)	47 (21.6%)	39 (24.5%)	8 (13.6%)	0.12	18 (31.6%)	29 (18.0%)	0.051
Calcium channel blockers (n; %)	26 (11.9%)	22 (13.8%)	4 (6.8%)	0.23	5 (8.8%)	21 (13.0%)	0.54
Diuretics (n; %)	15 (6.9%)	10 (6.3%)	5 (8.5%)	0.56	3 (5.3%)	12 (7.5%)	0.76
**Echo**							
LVEDD (mm)	46 (41, 50)	46 (41, 50)	46 (42.5, 50.25)	0.92	46 (39.5, 49.5)	46 (42, 50.25)	0.32
Mitral valve E Wave (m/s)	1.03 (0.85, 1.23)	1.02 (0.85, 1.23)	1.07 (0.84, 1.22)	0.63	1.1 (0.96, 1.27)	1.01 (0.80, 1.19)	0.023
Mitral valve A Wave (m/s)	0.60 (0.46, 0.73)	0.62 (0.48, 0.74)	0.57 (0.44, 0.69)	0.16	0.63 (0.47, 0.73)	0.60 (0.46, 0.73)	0.53
CoA maximum pressure gradient (mmHg)	32.52 (19.96, 45.10)	32.52 (19.68, 45.93)	33.00 (21.45, 44.51)	0.85	41.00 (30.26, 49.52)	29.04 (18.44, 42.61)	<0.01
CoA mean pressure gradient (mmHg)	16.45 (10.00, 22.39)	18.00 (10.73, 21.67)	13.21 (9.89, 27.19)	0.75	19.02 (16.00, 23.60)	15.00 (9.38, 21.80)	0.079
**MRI**							
LVEDV (mL/m^2^)	86.18 (77.67, 95.21)	84.42 (77.17, 94.13)	87.87 (79.53, 95.77)	0.17	86.51 (77.74, 93.35)	86.04 (77.33, 95.46)	0.99
LVESV (mL/m^2^)	32.93 (26.66, 40.50)	32.54 (25.75, 40.44)	35.35 (30.26, 40.96)	0.088	31.60 (24.05, 40.59)	33.15 (27.68, 40.30)	0.36
LV stroke volume (ml)	89.5 (69, 109.02)	90 (71.6, 111)	89 (62.7, 106)	0.29	85.31 (61.23, 104.5)	92.88 (72.05, 110.17)	0.093
LV mass (without papillary muscles) (g/m^2^)	53.05 (42.60, 64.13)	49.82 (42.16, 64.23)	54.33 (48.89, 61.31)	0.18	60.42 (46.93, 72.22)	50.48 (42.48, 58.97)	0.022
Ascending aorta distensibility	0.01 (0.00, 0.01)	0.01 (0.00, 0.01)	0.01 (0.00, 0.01)	0.15	0.01 (0.00, 0.01)	0.01 (0.00, 0.01)	0.34
Descending aorta distensibility	0.00 (0.00, 0.01)	0.00 (0.00, 0.01)	0.01 (0.00, 0.01)	0.45	0.00 (0.00, 0.01)	0.00 (0.00, 0.01)	0.64
Z-score of ascending aortic diameter	0.74 (−0.91, 3.01)	1.13 (−0.71, 3.07)	0.17 (−1.08, 1.67)	0.13	−0.30 (−1.39, 1.82)	1.13 (−0.24, 3.30)	0.013
Z-score of descending aortic diameter	1.85 (−0.04, 4.41)	2.79 (0.23, 4.99)	0.92 (−0.20, 2.75)	0.024	1.23 (−0.39, 4.19)	2.18 (0.23, 4.41)	0.24
**At-visit treatment decision**							
No treatment (n; %)	125 (57.3%)	92 (57.9%)	33 (55.9%)	0.92	31 (54.4%)	94 (58.4%)	0.71
Medication (n; %)	18 (8.3%)	13 (8.2%)	5 (8.5%)	1.00	3 (5.3%)	15 (9.3%)	0.41
Balloon angioplasty (n; %)	24 (11.0%)	18 (11.3%)	6 (10.2%)	1.00	7 (12.3%)	17 (10.6%)	0.91
Stenting (n; %)	45 (20.6%)	32 (20.1%)	13 (22.0%)	0.90	15 (26.3%)	30 (18.6%)	0.30
Surgery (n; %)	6 (2.8%)	4 (2.5%)	2 (3.4%)	0.66	1 (1.8%)	5 (3.1%)	1.00
**Post-visit outcome**							
Rehospitalization (n; %)	60 (27.5%)	47 (29.6%)	13 (22.0%)	0.35	56 (98.2%)	4 (2.5%)	<0.01
Difference between rehospitalization and visit (months)	24 (9.5, 42)	21.5 (8.25, 41.5)	26 (16.00, 42)	0.53	22 (9, 39.5)	43 (36.5, 48.5)	0.14
CoA-I (n; %)	57 (26.1%)	44 (27.7%)	13 (22.0%)	0.51	57 (100.0%)	0 (0.0%)	<0.01
Catheter-based intervention (n; %)	38 (17.4%)	28 (17.6%)	10 (16.9%)	1.00	38 (66.7%)	0 (0.0%)	<0.01
Catheterisation stenting (n; %)	22 (10.1%)	17 (10.7%)	5 (8.5%)	0.82	22 (38.6%)	0 (0.0%)	<0.01
Catheterisation balloon angioplasty (n; %)	32 (14.7%)	24 (15.1%)	8 (13.6%)	0.95	32 (56.1%)	0 (0.0%)	<0.01
Surgical reintervention (n; %)	27 (12.4%)	22 (13.8%)	5 (8.5%)	0.40	27 (47.4%)	0 (0.0%)	<0.01
CoA-S (n; %)	16 (7.3%)	12 (7.5%)	4 (6.8%)	1.00	16 (28.1%)	0 (0.0%)	<0.01
Difference between reintervention and visit (months)	14.5 (0.1, 49)	16 (0.1, 50)	5 (0.1, 39.5)	0.096	21.5 (8.50, 41.25)	10 (0.1, 50)	0.011
Death (n; %)	1 (0.5%)	1 (0.6%)	0 (0.0%)	1.00	0 (0.0%)	1 (0.6%)	1.00
Headache (n; %)	22 (10.1%)	18 (11.3%)	4 (6.8%)	0.46	11 (19.3%)	11 (6.8%)	0.015
Persistent arterial hypertension (n; %)	78 (35.8%)	66 (41.5%)	12 (20.3%)	<0.01	34 (59.6%)	44 (27.3%)	<0.01

Comparison of the development and validation datasets, as well as the CoA-I and non-CoA-I groups. Continuous data are expressed as median and interquartile range (IQR, Q1–Q3), with p-values derived from the Wilcoxon–Mann–Whitney test or independent t-test. Categorical data are presented as frequencies and percentages, with p-values obtained using Pearson's χ2 test.

Abbreviations: CoA-I, re-coarctation requiring intervention; CoA-S, re-coarctation requiring aortic surgery; AVD, aortic valve disease; BMI, body mass index; BSA, body surface area; CoA, coarctation of the aorta; LVEDD, left ventricular end-diastolic diameter; LVEDV, left ventricular end-diastolic volume; LVESV, left ventricular end-systolic volume; ACE, angiotensin-converting enzyme; LV, left ventricular.

**Fig. 1:**
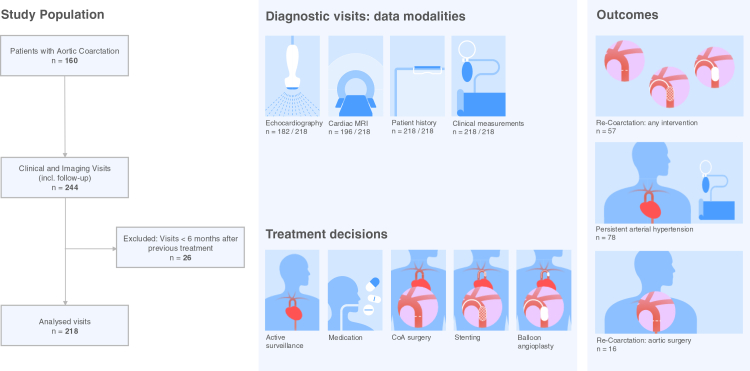
**Study design.** A visual summary of the study, including the formation of the study population (left panel), the parameters recorded during each visit, and possible treatment decisions (middle panel), followed by the future outcomes of interest (right panel).

At each visit, patients underwent echocardiography and/or CMR imaging, electrocardiography (ECG), and blood pressure measurements in both upper and lower extremities (middle panel, Fig. 1). The following data modalities were extracted from the hospital information systems, which contained a total of 788 features: baseline characteristics, medication intake, echocardiography measurements, CMR measurements, ECG measurements, intervention treatments, and post-visit outcomes (Table 1). Based on these examination results, a decision was made regarding the need for intervention at each visit.^16^ We defined guideline-based management using criteria in accordance with American College of Cardiology/American Heart Association (ACC/AHA) and European Society of Cardiology (ESC) guidelines.^13^^,^^17^ Heart catheterisation was recommended if (i) mean echo gradient >20 mmHg, (ii) mean echo gradient >10 mmHg with LVEF <40%, or (iii) evidence of arterial hypertension with a significant upper-to-lower extremity gradient (>20 mmHg). Further ESC-specific thresholds included a stenosis degree >50% alongside arterial hypertension.

Interventions were classified as invasive, including surgical reconstruction, stenting, and balloon angioplasty, or non-invasive, including antihypertensive medication or active surveillance. Post-visit outcomes were obtained through medical records, including outpatient reports (right panel, Fig. 1). CoA-I denoted any catheter-based or surgical reintervention during follow-up, whereas CoA-S denoted the surgical subset of CoA-I. For the definition of hypertension, blood pressure percentiles were calculated according to age, sex, and height for patients aged under 18 years.^18^ For those older than 18 years, an approach accounting for age and body mass index (BMI) was used.^19^

### Ethics statement

The study adheres to the Transparent Reporting of a multivariable prediction model for Individual Prognosis Or Diagnosis (TRIPOD) checklist^20^ and the Strengthening the Reporting of Observational Studies in Epidemiology (STROBE; Supplementary Tables S1–S2) statement.^21^ The study was approved by the institutional ethics committee (Charité–Universitätsmedizin Berlin: EA2/172/13) and conducted in accordance with the principles of the Declaration of Helsinki. All participants or their legal representatives provided written informed consent before enrolment.

### Imaging

Transthoracic echocardiography was performed in all patients using a standard ultrasound system (e.g., Vivid E9, GE HealthCare). In adolescents and adults, a 3.5 MHz phased-array transducer was typically employed, whereas higher-frequency paediatric probes (5–8 MHz) were used in younger children. This approach aligns with current American Society of Echocardiography/European Association of Cardiovascular Imaging (ASE/EACVI) guidelines and ensures optimal image resolution across age groups. Two-dimensional images of the left and right ventricles were obtained from the parasternal (long- and short-axis), apical (four-, two-, and three-chamber), and subcostal views to assess chamber dimensions and global systolic function. Pulsed-wave (PW) and continuous-wave (CW) Doppler measurements were performed at the valves, as well as in the left and right ventricular outflow tracts. Additionally, CW Doppler assessment of the aortic isthmus was conducted via the suprasternal or high-right parasternal window to measure peak systolic velocity and derive the corresponding pressure gradient. Each measurement was averaged over at least three consecutive cardiac cycles.

CMR examinations were performed on a 1.5 T Philips Achieva R 3.2.2.0 system with a five-element cardiac phased-array coil. Imaging followed a standardised protocol, including balanced Turbo Field Echo (bTFE) cine in consecutive short-axis slices for left ventricular volumetry and anatomical measurements. Cross-sectional diameters (aortic valve annulus, coronary aortic sinus, sinotubular junction, ascending aorta, descending aorta, aortic arch, and isthmus) were normalised to body surface area (BSA) and converted to Z-scores.^22^ Two-dimensional velocity-encoded CMR was captured distal to the aortic valve to assess effective antegrade flow, stroke volume, cardiac index, and cardiac output. Typical parameters for velocity-encoded sequences were voxel size 2 × 2 × 5 mm^3^ (reconstructed to 1 × 1 × 5 mm^3^), echo time 4 ms, repetition time 3 ms, flip angle 15°, 30 automatically reconstructed phases, and retrospective cardiac gating. For bTFE cine, voxel size was 1.80 × 1.70 × 6 mm (reconstructed to 1 × 1 × 6 mm), echo time 1.2 ms, repetition time 2.5 ms, flip angle 60°, and 40 reconstructed phases, requiring ∼9–14 min of scan time.

### Machine learning

**Fig. 2:**
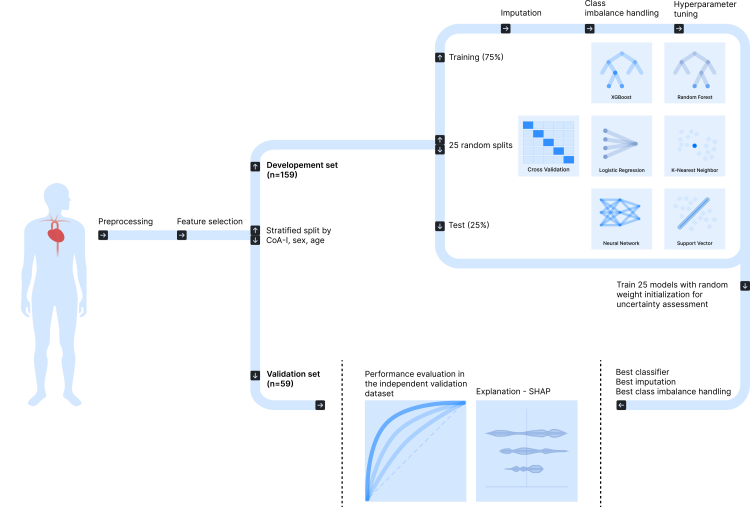
**Machine learning pipeline.** A visual summary of the machine learning pipeline implemented in the study. Following data preprocessing and feature preselection, the data were split into development and validation datasets. Within the development set, 25 random splits were used to select the optimal classifier, imputation strategy, and class imbalance handling. The final model was then retrained on the entire development set using 25 random initialisations. Performance and SHAP analysis were evaluated in the validation set. Abbreviations: SHAP, SHapley Additive exPlanations; CoA-I, re-coarctation requiring intervention.

A summary of the ML pipeline is illustrated in Fig. 2. Prior to model development, data preprocessing and feature selection were performed (Supplementary Methods). To mitigate the impact of dataset splits on model performance, 25 random splits (75:25 ratio) within the development set were employed. Since most ML models cannot directly handle missing values, imputation was employed within the training data of each split/fold. Binary features with missing values were replaced by the most frequent class, and numeric features were replaced by the median. Following imputation, all features were normalised to a range between 0 and 1, using scaling parameters derived from the respective training data. Feature-wise missingness was modelled as a function of observed clinical variables (Supplementary Table S3, Supplementary Figure S1). As a sensitivity analysis, mean and iterative random-forest imputation were additionally compared with median imputation (Supplementary Table S4). To address class imbalance during model training, where the number of cases and controls was unequal, BorderlineSMOTE from the Python package imblearn (v0.12.0) was used to generate synthetic positive cases for balancing the data in the development dataset, within the respective training split/fold only. In sensitivity analyses, class-weighted learning without synthetic upsampling was also evaluated, and the better-performing strategy for each outcome was retained for final validation (Supplementary Tables S4–S5).

Hyperparameters were tuned using Bayesian search with 5-fold cross-validation in the development dataset, with optimisation confined to the respective training folds (Supplementary Methods and Supplementary Table S6). Seven classifiers were evaluated, including CatBoost, XGBoost, random forest, support vector classifier with a radial kernel, neural network, logistic regression, and K-nearest neighbours. Classifiers were implemented using the Python (v3.11) scikit-learn package (v1.4.1), XGBoost (xgboost, v2.0.3) and CatBoost (catboost, v1.2.3).

For uncertainty assessment, the best-performing classifier for each outcome, together with the selected imputation and class-imbalance handling strategy, was retrained 25 times on the entire development dataset using different random seeds. Validation performance and its uncertainty were subsequently assessed in the independent validation dataset. To enhance the interpretability of the ML models, Shapley Additive exPlanations (SHAP) values were used to rank features by their impact and to indicate the direction and magnitude of their effect on the best-performing classifier's predictions.

### Feature interactions

To assess the impact of feature interactions on the ML model's performance and determine whether they provide additional predictive information, feature pairs were identified by training a CatBoost classifier with optimised hyperparameters and a tree depth of two. The selection process entailed the first 20 base learners, as the initial base learners capture most of the variance in the model's predictions. The identified feature pairs were then transformed into interaction terms by multiplying their values and incorporated into the final model training alongside the existing pool of 38 features.

### Treatment effects model

Cohort-based treatment effects on CoA-I and future hypertension were evaluated using inverse-probability weighting (IPW) as the primary approach and propensity-score matching as a sensitivity analysis. The weighted analysis used index-visit treatment as a multicategory exposure and a multinomial logistic treatment-assignment model including age, previous stenting, previous balloon angioplasty, and previous aortic surgery to estimate average treatment effects across treatment categories. Detailed treatment-assignment model coefficients and covariate-balance information before and after weighting/matching are provided in the Supplementary Methods and Supplementary Tables S7–S10.

### Statistical analysis

To evaluate model discrimination, we used the area under the receiver operating characteristic curve (ROC AUC) as the primary metric, and additionally reported the area under the precision–recall curve (PR AUC), which was interpreted relative to the baseline prevalence of positive cases. Additional evaluation measures included true positives (TP), true negatives (TN), false positives (FP), and false negatives (FN), from which standard performance metrics were derived: accuracy = (TP + TN)/(TP + FP + TN + FN), precision = TP/(TP + FP), recall (or sensitivity) = TP/(TP + FN), specificity = TN/(TN + FP), F1-score = 2 × (precision × recall)/(precision + recall), and negative predictive value (NPV) = TN/(TN + FN). To minimise sampling bias, visits were only included when spaced by at least six months, and class imbalance was handled within the training data during model fitting.

Continuous data (unless stated otherwise) are expressed as median and interquartile range (IQR, Q1–Q3), while categorical data are presented as frequencies and percentages. A p-value < 0.05 was considered statistically significant. Data were tested for normal distribution using the Shapiro–Wilk test. An independent-sample t-test or Wilcoxon–Mann–Whitney test was used for continuous group comparisons. For performance comparison across the 25 repeated splits, paired differences in ROC AUC were analysed using either a paired t-test or Wilcoxon signed-rank test, as appropriate. Pearson's χ2 test and Fisher's exact test were used for comparisons of categorical variables. As a supplementary time-to-event analysis, a Cox proportional hazards model was fitted for time to CoA-I after the index visit (Supplementary Methods). All statistical analyses were performed using Stata (19.0 MP) and SPSS (29.0).

### Web calculator

A web-based calculator (Flask v3.1.0, onnxruntime v1.20.1) was created to support reproducibility and exploratory use. The calculator integrates the outputs of the best-performing ML classifiers using structured clinical and imaging variables selected from the SHAP analysis. Missing values are automatically imputed, allowing partial data entry. The displayed readouts comprise model-based risk estimates generated by the final trained classifiers. Alongside model-based risk estimates, the calculator displays separate guideline-based reference thresholds derived from the AHA/ACC and ESC criteria, providing additional clinical context.^13^^,^^17^ The tool is provided as an open research interface for reproducibility and external validation.

### Role of the funding source

The funder of the study had no role in study design, data collection, data analysis, data interpretation, or writing of the report.

## Results

### Patient characteristics and cohort distribution

A total of 160 patients with CoA were included in the study. Multiple follow-up visits resulted in a final dataset of 218 index visits (Fig. 1, left panel) with a total of 196 CMR examinations. The median patient age was 18 years (IQR: 13–28) and the median follow-up time was 45.5 months (IQR: 17–67). The data were split into a training cohort (n = 159) and a separate prespecified validation cohort (n = 59), ensuring similar distributions for CoA-I, age, and sex for ML model validation (Table 1).

Table 1 shows that most index-visit features, including treatment decisions, were comparable between the development and validation cohorts (p > 0.05). The dataset exhibited significant class imbalance, with 26% of cases for CoA-I (n = 57), 7% for CoA-S (n = 16) as the surgical subset of CoA-I, and 36% for hypertension (n = 78). Features associated with CoA-I (p < 0.05) included age, BMI, BSA, aortic valve disease (AVD) with isolated aortic regurgitation, mitral valve disease, maximum pressure gradient, and post-visit outcomes, such as rehospitalisation, headache, and persistent arterial hypertension. In addition to outcome prediction, ML models were also trained to assess treatment decisions at the index visit, distinguishing between invasive intervention (balloon angioplasty, stenting, or surgery) and a non-invasive approach (active surveillance or medical intervention).

### Model performance

**Fig. 3:**
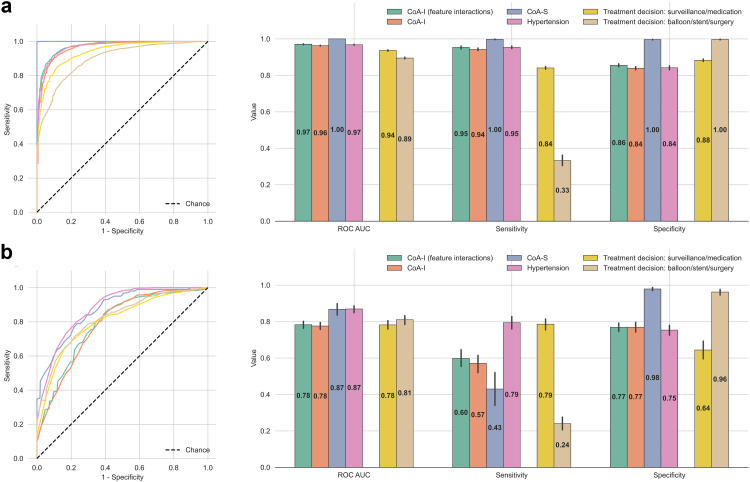
**Model performance in the development dataset.** Performance metrics obtained from 25 random stratified train–test splits within the development dataset, shown for (a) the training and (b) the test subsets. A distinct best-performing classifier was selected for each outcome (columns 1–4) and treatment decision (columns 5–6). For CoA-I, two classifiers were compared, one incorporating feature interactions and the other using only single features. The left panel shows the mean ROC curves, while the right panel includes bar plots showing the mean ROC AUC, sensitivity, and specificity, with error bars indicating the 95% CI. Abbreviations: CoA-I, re-coarctation requiring intervention; CoA-S, re-coarctation requiring aortic surgery; ROC, receiver operating characteristics; AUC, area under the curve; CI, confidence interval.

Performance metrics of all ML algorithms during model development are summarised in Supplementary Table S11. Fig. 3 shows the development performance metrics of the best-performing classifiers selected according to the ROC AUC. CatBoost yielded the highest ROC AUC (0.78 ± 0.05) for CoA-I and persistent arterial hypertension (0.87 ± 0.05), whereas the support vector classifier performed best for CoA-S (0.87 ± 0.09).

Within the prespecified validation cohort (Table 2), a ROC AUC of 0.90 ± 0.01 was achieved for the prediction of CoA-S, as well as CoA-I when incorporating feature interactions. Prediction of CoA-I without interaction terms yielded slightly lower performance, with a ROC AUC of 0.89 ± 0.01. The model for predicting persistent arterial hypertension attained a ROC AUC of 0.84 ± 0.01.

**Table 2:** Performance metrics of the best-performing machine learning classifier on development and validation datasets.

		F1		ROC AUC		Sensitivity		Specificity		Accuracy		Precision		NPV		PR AUC	
**Post-visit outcome**	Model	Dev	Val	Dev	Val	Dev	Val	Dev	Val	Dev	Val	Dev	Val	Dev	Val	Dev	Val
CoA-I (feature interactions)	cat	0.90 (0.01)	0.75 (0.02)	0.96 (0.00)	0.90 (0.01)	0.94 (0.01)	0.90 (0.04)	0.86 (0.02)	0.75 (0.03)	0.90 (0.01)	0.79 (0.02)	0.87 (0.02)	0.51 (0.03)	0.93 (0.01)	0.96 (0.01)	0.96 (0.01)	0.69 (0.06)
CoA-I	cat	0.90 (0.01)	0.75 (0.02)	0.95 (0.00)	0.89 (0.01)	0.93 (0.02)	0.82 (0.04)	0.87 (0.02)	0.79 (0.03)	0.90 (0.01)	0.79 (0.02)	0.87 (0.02)	0.52 (0.03)	0.92 (0.01)	0.94 (0.01)	0.95 (0.01)	0.64 (0.06)
CoA-S	svc	1.00 (0.00)	0.78 (0.02)	1.00 (0.00)	0.90 (0.01)	1.00 (0.00)	0.75 (0.00)	0.99 (0.00)	0.94 (0.01)	1.00 (0.00)	0.93 (0.01)	0.99 (0.00)	0.49 (0.04)	1.00 (0.00)	0.98 (0.00)	1.00 (0.00)	0.63 (0.05)
Persistent arterial hypertension	cat	0.91 (0.01)	0.71 (0.02)	0.97 (0.00)	0.84 (0.01)	0.92 (0.02)	0.78 (0.04)	0.90 (0.02)	0.77 (0.02)	0.91 (0.01)	0.77 (0.01)	0.86 (0.02)	0.46 (0.02)	0.94 (0.01)	0.94 (0.02)	0.97 (0.00)	0.46 (0.01)
**Index-visit treatment decision**																	
Active surveillance/medication only	xgb	0.84 (0.01)	0.80 (0.03)	0.92 (0.00)	0.86 (0.02)	0.83 (0.01)	0.90 (0.03)	0.84 (0.02)	0.68 (0.05)	0.84 (0.01)	0.82 (0.03)	0.84 (0.02)	0.84 (0.02)	0.83 (0.01)	0.80 (0.06)	0.93 (0.00)	0.89 (0.02)
Balloon angioplasty/stenting/surgery	xgb	0.81 (0.01)	0.77 (0.02)	0.91 (0.00)	0.87 (0.01)	0.68 (0.02)	0.57 (0.04)	0.92 (0.02)	0.94 (0.02)	0.84 (0.01)	0.81 (0.02)	0.82 (0.03)	0.84 (0.04)	0.85 (0.01)	0.80 (0.01)	0.87 (0.01)	0.82 (0.02)

The performance of the best-performing classifier on the training and validation cohorts, determined using a stratified split based on sex, age, and CoA-I. Metrics are reported as mean values, with standard deviations shown in parentheses.

Abbreviations: CoA-I, re-coarctation requiring intervention; CoA-S, re-coarctation requiring aortic surgery; ROC, receiver operating characteristics; PR, precision-recall; AUC, area under the curve; NPV, negative predictive value; cat, CatBoost; xgb, XGBoost; svc, support vector classifier; dev, development dataset; val, validation dataset.

Missingness was predictable from observed covariates, supporting the plausibility of a Missing at Random (MAR) rather than a Missing Completely At Random (MCAR) concept (Supplementary Table S3). Alternative imputation strategies did not materially improve performance (Supplementary Tables S4–S5) and median imputation was therefore retained for the final models.

Sensitivity analyses comparing BorderlineSMOTE with class-weighted learning showed no significant differences for most outcomes. The persistent arterial hypertension and one of the index-visit treatment decision models performed better without upsampling (mean ROC AUC improvement 0.013, p < 0.01 and 0.021, p < 0.001, respectively); these models were therefore retrained using class-weighted learning (without upsampling) and revalidated (Table 2, Fig. 3, Supplementary Table S5). PR AUC and threshold-based metrics are additionally reported in Table 2 and Supplementary Table S11. In additional sensitivity analyses, performance improved when visits from patients also represented in the development dataset were excluded (n = 24) and worsened when only such visits were retained (CoA-I ROC AUC 0.95 and 0.84 versus 0.90 in the main analysis; Supplementary Tables S12–S13).

### Feature importance based on Shapley additive explanations

SHAP were used to interpret the final optimised models. The best-performing classifiers, as determined by the highest ROC AUC in the preselected validation cohort (Table 2), were selected for SHAP analysis to gain deeper insights into model predictions. SHAP provides a unified framework for quantifying feature importance, offering insights into how individual features influence model predictions by fairly distributing the output contribution among input variables based on cooperative game theory.^23^

**Fig. 4:**
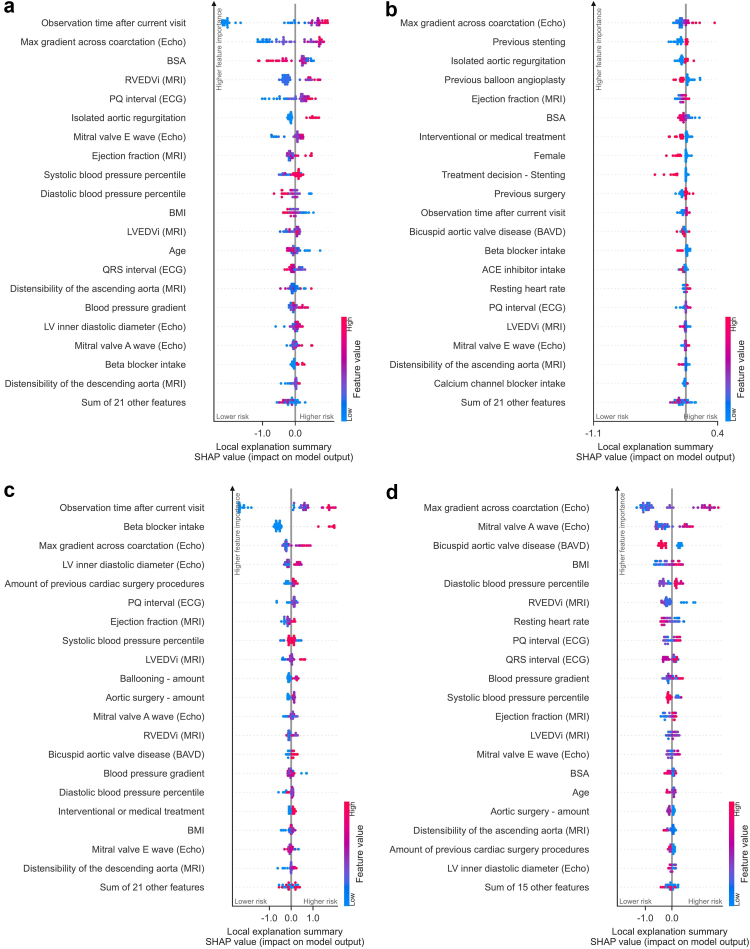
**Feature importance for outcome and treatment prediction based on SHAP analysis.** Top 20 features ranked by mean absolute SHAP values, indicating their global importance in predicting CoA-I without feature interactions (a), CoA-S (b), persistent arterial hypertension (c), and invasive intervention at the index visit (d). The colour scale (blue to red) represents the feature values, with continuous variables shown as a gradient and binary variables as discrete colours (blue for 0, red for 1). Each dot corresponds to an individual case, and the horizontal position reflects the feature's contribution to the model's prediction. The vertical line at zero represents the baseline; dots farther from this line indicate a greater impact on the prediction. Positive SHAP values (right from baseline) increase the predicted probability of the outcome, while negative values (left from baseline) decrease it. Abbreviations: CoA-I, re-coarctation requiring intervention; CoA-S, re-coarctation requiring aortic surgery; SHAP, SHapley Additive exPlanations; LVEDVi, left ventricular end-diastolic volume index; RVEDVi, right ventricular end-diastolic volume index; BSA, body surface area; BMI, body mass index; LV, left ventricular; ACE, angiotensin-converting enzyme.

Fig. 4 presents SHAP values along with the individual impacts of observations for each feature. For CoA-I prediction without interaction terms (Fig. 4a), a longer observation time after the index visit emerged as the most important feature. Higher values of maximum gradient across the coarctation, right/left ventricular end-diastolic volume index (RVEDVi, LVEDVi), PQ interval, mitral valve E wave, systolic blood pressure percentile, and left ventricular end-diastolic diameter were associated with an increased CoA-I risk. Conversely, higher values of BSA, BMI, age, and QRS duration were linked to a lower likelihood of CoA-I. Additionally, a diagnosis of aortic regurgitation increased the CoA-I risk.

Several feature interactions exhibited high absolute SHAP values (Supplementary Figure 2a), with observation time after the index visit being a common component for CoA-I prediction. The most predictive interaction terms included observation time after the index visit, multiplied by BSA and by maximum gradient across the coarctation. Current or past treatments were not identified as strong predictors of increased CoA-I risk.

In the CoA-S SHAP summary (Fig. 4b), the maximum gradient across the stenosis showed the largest absolute SHAP values. Among treatment-related variables, prior stenting or surgery and absence of prior balloon angioplasty were associated with positive SHAP values, whereas current treatment, particularly stenting at the index visit, was associated with negative SHAP values. For persistent arterial hypertension (Fig. 4c), the most influential predictors were longer observation time after the index visit, beta-blocker intake, and a high gradient across the coarctation.

### Patient-level interpretability

**Fig. 5:**
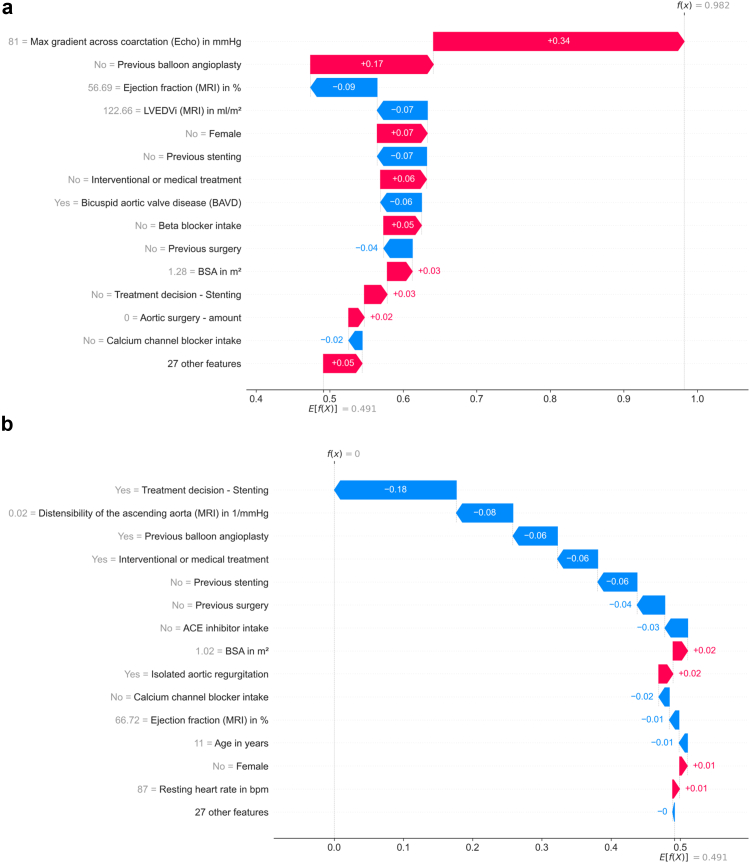
**SHAP waterfall analysis of high- and low-risk CoA-S cases.** SHAP values for the 15 most important features are visualised for two test cases with high (a) and low (b) predicted probabilities of CoA-S based on the SVC model. The cumulative SHAP score for each observation is denoted as f(x), while E[f(X)] represents the base value (the model's expected output across all instances). Red bars indicate features increasing the probability of CoA-S, while blue bars represent features decreasing the risk. Feature values for each case are displayed in grey to the left of the equal sign. Abbreviations: CoA-S, re-coarctation requiring aortic surgery; SVC, support vector classifier; SHAP, SHapley Additive exPlanations; ACE, angiotensin-converting enzyme; LVEDVi, left ventricular end-diastolic volume index; BSA, body surface area.

Local SHAP values quantify the contribution of individual features to the model output for a specific instance. Fig. 5 illustrates two exemplified cases from the validation cohort for CoA-S after guideline-based treatment decisions at their index visit, one with a high predicted risk of 98.2% (Fig. 5a) and another with a low predicted risk of 0.0% (Fig. 5b). In the high-risk case, the strongest contributing factors in this 14-year-old male patient, who was not a suitable candidate for a stenting procedure, were a high gradient across the stenosis (81 mmHg) and the absence of current treatment options or prior interventional balloon angioplasty (Fig. 5a). Functional and anthropometric data, as well as other previous treatment information, showed a mixed effect. In contrast, no single feature emerged as a primary protective factor for the low-risk case of an 11-year-old boy (Fig. 5b). Instead, several contributing factors collectively lowered the probability of CoA-S. These included the presence of interventional or medical treatment (especially stenting) at the index visit, and the absence of previous interventions, including surgery and stenting.

### Treatment decision and treatment effects

Models were furthermore trained to classify whether patients received an invasive or non-invasive approach at the index visit. XGBoost trained on random splits achieved a ROC AUC of 0.78 ± 0.06 for non-invasive treatment and 0.81 ± 0.06 for invasive intervention (Supplementary Tables S4, S11). In an additional fixed-split model (Table 2), the performance of both models improved to a ROC AUC of 0.86 ± 0.02 and 0.87 ± 0.01, respectively. Sensitivity analyses favoured class-weighted learning without synthetic upsampling only for one of the index-visit treatment-decision models (Supplementary Tables S4–S5). As illustrated in Fig. 4d, higher values of the gradient across the stenosis, mitral valve A wave, BMI, and diastolic blood pressure were indicative of invasive treatment at the index visit.

Cohort-based treatment effects on CoA-I and hypertension were evaluated using IPW as the main analysis and propensity-score matching as a sensitivity analysis. For the composite outcome CoA-I, antihypertensive medication reduced the likelihood of CoA-I by 17.3% (95% confidence interval [CI], −28.2 to −6.4; p = 0.002). Balloon angioplasty (−3.0%; 95% CI, −19.4 to 13.4; p = 0.717), stenting (7.2%; 95% CI, −8.3 to 22.6; p = 0.361), and surgery (−11.4%; 95% CI, −33.7 to 11.0; p = 0.319) were not statistically significant. In propensity-score-matched sensitivity analyses, the medication estimate remained concordant (−17.3%; 95% CI, −31.8 to −2.8; p = 0.019), while balloon angioplasty (−8.4%; 95% CI, −25.7 to 8.9; p = 0.341) and stenting (1.6%; 95% CI, −13.5 to 16.6; p = 0.838) were not statistically significant.

For the haemodynamic outcome of future hypertension, medication (−4.0%; 95% CI, −21.9 to 13.9; p = 0.663) and balloon angioplasty (2.2%; 95% CI, −16.3 to 20.7; p = 0.816) were not statistically significant in IPW analyses, whereas stenting (17.4%; 95% CI, 0.5–34.3; p = 0.044) and surgery (42.5%; 95% CI, 11.4 to 73.6; p = 0.007) were associated with a higher probability of future hypertension. The matched sensitivity analyses were not statistically significant for medication (−2.8%; 95% CI, −31.8 to 26.2; p = 0.850), balloon angioplasty (10.1%; 95% CI, −12.9 to 33.0; p = 0.390), and stenting (8.7%; 95% CI, −15.2 to 32.6; p = 0.474). A separate matched surgical analysis was not performed because only six surgical visits were available. A supplementary Cox proportional hazards analysis for time to CoA-I after the index visit is shown in Supplementary Figure S3.

### Web calculator

The web-based calculator implementing the final model weights with a reduced feature set is available at https://icm.dhzc.charite.de/calc_coa and displays interactive plots of predicted outcome probabilities over time (Supplementary Figures S4–S5). This implementation showed no relevant loss of performance in sensitivity analyses (mean ROC AUC difference [reduced minus default] −0.008 to 0.001; p = 0.10–0.79; Supplementary Table S4). Additional user instructions, limitations, and data-handling information are provided in the Supplementary Material and in an embedded tutorial video.

## Discussion

Our study demonstrates that data-driven ML models robustly predict the risk of persistent arterial hypertension and re-coarctation requiring intervention (CoA-I) or surgery (CoA-S) among patients with CoA, using routine clinical data and multimodal imaging assessments (echocardiography and CMR). Beyond cohort-derived group-level estimates, the models generate patient-specific probabilities. A web-based research interface reproduces these outputs alongside rule-based guideline thresholds and predicted treatment likelihood, providing a practical platform for future prospective multicentre evaluation.

Furthermore, feature importance analysis clarified how specific risk factors contribute, aligning with previous evidence linking structural and functional markers, including imaging markers and aortic wall characteristics, to adverse outcomes in CoA.^17^^,^^24^^,^^25^ Our findings suggest that previously less-emphasised echocardiographic parameters, ECG variables, and CMR-derived indexed volumetric measures may provide additional information when considered jointly within a multimodal explainable ML framework rather than in isolation.

Hypertension is a well-established risk factor for long-term morbidity in patients with CoA, even after successful repair.^4^^,^^26^^,^^27^ In our study, elevated systolic blood pressure, an increased brachial-ankle pressure gradient, and a higher Doppler-derived pressure gradient across the coarctation site were all associated with an increased likelihood of reintervention, despite patients having been managed in accordance with current clinical guidelines at the index visit.^17^ These findings underscore that, even under guideline-directed management, residual haemodynamic burden in higher-degree stenosis may persist and contribute to the future need for reintervention. While pharmacological therapy would typically be expected to mitigate hypertension risk, an association between beta-blockers and hypertension likely reflects confounding by indication, with the drug preferentially prescribed to patients already diagnosed with hypertension.

Furthermore, the time elapsed after the index visit, together with BSA and other anatomical measures, contributed significantly to CoA-I and arterial hypertension risk. This resonates with the concept that ongoing somatic growth, particularly in paediatric and adolescent populations, alters aortic wall properties and haemodynamics^3^^,^^26^^,^^28^ and concurs with previous evidence linking prolonged exposure to haemodynamic stress to vascular remodelling.^29^ Imaging-based parameters, including RVEDVi on CMR and mitral valve E wave on echocardiography, were associated with a higher risk of CoA-I. These features can be indicative of elevated left ventricular filling pressure or left ventricular hypertrophy, which serves as an important marker of disease progression.^17^^,^^30^

When the most important feature interactions were incorporated into the model predicting CoA-I, performance improved, and several top feature pairs were identified in the SHAP plot: Combining time after the index visit with BSA, maximum gradient across the coarctation, and systolic blood pressure showed a strong predictive value. Although the clinical significance of these interactions is not yet fully established, their inclusion demonstrably improved model performance, suggesting a potential synergistic effect beyond individual variable contributions.

Our findings mainly portray cases where decisions were made in accordance with established clinical guidelines and borderline cases, thereby limiting extrapolation to clinical settings where management deviates substantially from recommended guidelines.^31^ Additionally, the distinction between catheter-based reintervention and surgery may still partly reflect institutional practice and provider-specific factors not captured. In this mixed population of previously treated and native CoA, with previously treated cases forming the larger subgroup, treatment choice at the index visit contributed limited additional information to the prediction of future CoA-I, likely reflecting the relatively uniform adherence to guideline-based treatment criteria and a narrow range of residual haemodynamic burden after the index visit.^13^^,^^17^ Nevertheless, additional IPW and propensity-based analyses of the entire dataset indicated a cohort-level association between antihypertensive medication and a reduced risk of CoA-I. This finding aligns with the established benefit of stringent blood pressure control in patients with CoA.^4^^,^^27^ By contrast, the analyses concerning balloon angioplasty and stenting remained inconclusive, highlighting a differential clarity of treatment effects predominantly evident for antihypertensive therapy in patients in whom invasive treatment was not indicated.

By displaying current guideline thresholds together with model-derived patient-specific probabilities and estimated treatment likelihood, the online platform complements guideline-based assessment with individualised probabilistic information. The added clinical value of this approach requires prospective evaluation, and it is not intended to replace clinical judgement or to serve as the sole basis for individual patient management. Future integration of patient-specific computational fluid dynamics (CFD) or structural models may further optimise intervention planning.^31^, ^32^, ^33^

Despite the promising findings, several limitations should be acknowledged. Although this was a single-centre study, the cohort reflects a realistic routine-care population and a hold-out test set mitigated the risk of overfitting. However, external validity in centres with substantially different case mixes may be limited. Accordingly, prospective validation in diverse multicentre cohorts remains essential to confirm the model's generalisability. The accompanying web tool already enables investigators at different institutions to apply the model to their own datasets, thereby facilitating independent validation. We intentionally treated each follow-up visit as an independent case to capture a variety of patient-specific profiles that included somatic growth, anatomical changes and evolving haemodynamics. This approach allowed us to reflect the diversity of patient profiles, which can change substantially over time, even within the same individual. As analyses were conducted at the visit level, repeated observations from the same patient may still introduce some dependence. To mitigate this, we excluded visits that were separated by fewer than six months to avoid over-representing patients with largely unchanged clinical status or carry-over treatment effects attributable to a closely preceding intervention. While this strategy may have omitted some relevant short-term change in patient-specific profiles, it helped to preserve the focus on clinically meaningful variation over time. In additional sensitivity analyses, performance improved when overlapping-patient validation visits were excluded and worsened when only such visits were retained, suggesting that repeated visits often represented distinct clinical states rather than simple patient-specific carry-over effects. This cohort included both previously treated and native CoA, but was weighted toward post-interventional follow-up; accordingly, conclusions regarding untreated native CoA at first presentation should be interpreted in the context of this cohort composition. Furthermore, the study interval varied across individuals, and late complications might therefore be underestimated.

Another limitation is the use of data imputation techniques. While imputation can help mitigate the impact of missing data, it may also introduce errors, potentially affecting the accuracy of the ML models. Notably, a substantial proportion of our cohort had comprehensive imaging data from CMR and echocardiography, along with standard clinical parameters such as blood pressure gradients and anthropometrics, thereby reducing the extent of missing values. Throughout model training and development, imputation was applied to the training cohort, in line with standard practice to minimise data leakage. Furthermore, we employed algorithms such as CatBoost, which natively handle missing data. Although the overall dataset comprised 218 visits, splitting into development and validation cohorts particularly affected the low-frequency outcome CoA-S (n = 16), reducing its effective sample size and increasing estimate uncertainty. Smaller datasets inherently increase the risk of overfitting, as they may lack the necessary variability to ensure successful generalisation. Despite these constraints, the test set findings demonstrate that routinely collected clinical and imaging data can yield robust models that accurately predict the outcome.

Our web-based interface illustrates how a flexible decision-support platform can aid clinicians in applying these ML predictions. Researchers can deploy the risk calculator and evaluate its predictive accuracy against clinical outcomes at their own centres. This facilitates decentralised validation, promoting methodological transparency and supporting external benchmarking across institutions. Over time, if multicentre collaboration enables sharing of harmonised, de-identified data, external validation may become more seamless, further enhancing the generalisability of the approach.

This study underscores the potential of ML models for predicting arterial hypertension and re-coarctation risks in patients with CoA using data from routine clinical and imaging sources. The web-based risk calculator enables healthcare professionals to identify high-risk individuals and consider timely diagnostic or therapeutic adjustments. The use of structured reporting of echocardiographic and CMR data, in conjunction with the computational efficiency of the tree-based model, which requires less than 1 min to train, makes it feasible to retrain the model as new data become available. This opens the door to continuous model refinement and enables the integration of updated versions into the web-based risk calculator. Although further validation in broader populations is essential, these findings mark a step towards patient-tailored follow-up strategies in CoA, aiming to reduce lifetime cardiovascular complications and improve long-term outcomes.

## Contributors

L.F. reviewed the clinical data, conducted the statistical analyses, generated the figures, and drafted the initial version of the manuscript. J.V. performed the machine learning analyses, developed and maintained the web-based risk calculator, contributed to figure generation, and co-drafted the manuscript. L.F., J.V., and M.K. accessed and verified the data. G.G. provided technical support in deploying the web calculator. T.K. and M.K. coordinated the study, revised the manuscript, and conducted the propensity score analysis. G.M. contributed to the design and implementation of interpretable machine learning methods. P.K., L.G., and F.B. critically reviewed the final version of the manuscript. All authors reviewed and approved the final draft.

## Data sharing statement

Data from this study will be shared upon reasonable request to the corresponding author, provided that such sharing complies with applicable data protection regulations. Data access can only be granted within the framework of data protection requirements, including appropriate confidentiality agreements and ethical approvals where necessary.

## Declaration of interests

All authors have no conflicts of interest to disclose.
