# Supplementary material for: Prediction of hypertension and restenosis under guideline-directed management in aortic coarctation: development and validation of machine-learning models
Source: eClinicalMedicine. 2026 Jun 26;97:104041. doi: 10.1016/j.eclinm.2026.104041 (PMC13320318; doi:10.1016/j.eclinm.2026.104041)
Supplement: Supplementary Materials [file mmc1.pdf]

Supplementary Material

Supplementary Methods

Supplementary Figure

Supplementary Figure 1. Missing data across modalities

Supplementary Figure 2: Feature importance for outcome and treatment prediction based on SHAP analysis

Supplementary Figure 3. Hazard ratios for CoA-I

Supplementary Figure 4: Web calculator interface

Supplementary Figure 5. Mean absolute SHAP value of features included in the reduced models

Supplementary Tables

Supplementary Table 1: TRIPOD checklist

Supplementary Table 2: STROBE checklist

Supplementary Table 3: Predictability of missingness from observed variables

Supplementary Table 4: Sensitivity analyses in the development dataset

Supplementary Table 5: Final model selection used for validation

Supplementary Table 6 Optimised hyperparameters per classifier

Supplementary Table 7A: Endpoint-specific treatment-effect estimates for CoA-I

Supplementary Table 7B: Endpoint-specific treatment-effect estimates for future hypertension

Supplementary Table 8A: Multinomial treatment-assignment model shared by the inverse-probability-weighted analyses of CoA-I and future hypertension

Supplementary Table 8B: Binary propensity-score models shared by the matched sensitivity analyses of CoA-I and future hypertension

Supplementary Table 9A: Effective weighted group sizes for the inverse-probability-weighted analyses of CoA-I and future hypertension

Supplementary Table 9B: Baseline group summaries for the inverse-probability-weighted analyses of CoA-I and future hypertension

Supplementary Table 9C: Post-weighting balance diagnostics for the inverse-probability-weighted analyses of CoA-I and future hypertension

Supplementary Table 10A: Restricted and matched sample sizes for the propensity-score sensitivity analyses of CoA-I and future hypertension

Supplementary Table 10B. Baseline group summaries for the propensity-score sensitivity analyses of CoA-I and future hypertension

Supplementary Table 10C. Post-matching balance diagnostics for the propensity-score sensitivity analyses of CoA-I and future hypertension

Supplementary Table 11: Performance comparison of ML classifiers for outcome and treatment prediction

Supplementary Table 12: Performance metrics in the validation dataset after excluding visits from patients who appeared in both the development and validation datasets

Supplementary Table 13: Performance metrics in the validation dataset including only visits from patients who appeared in both the development and validation datasets

Supplementary Methods

ML Preprocessing

Binomial, categorical, and numerical features were retained; text and date features were discarded. Categorical variables were converted via one-hot encoding. Features with more than 35% missing values were removed, except for clinically pivotal ascending/descending aortic distensibility and right ventricular end-diastolic volume index (RVEDVi) with 48%, 50%, and 48% missing values, respectively. Numeric features with variance below 0.005 or correlation above 0.90 were excluded. Binary features with fewer than 2% positive instances were excluded. This initial preselection yielded 33 binary, 4 categorical, and 42 numeric features. The list was further reviewed to include only clinically relevant features, resulting in the final set of 38 features (41 after one-hot encoding was applied) used for model development.

Predicting missingness based on observed variables

To provide evidence against Missing Completely At Random (MCAR), we modelled feature-wise missingness as a function of observed covariates. Missing at random (MAR) cannot be proven from observed data alone. However, if missingness is predictable from observed variables, MCAR is unlikely and MAR becomes plausible. For each feature with >10% missing values, we defined a binary indicator (missing vs. observed) and fitted a logistic regression model using age, sex, CoA-I status, treatment decision, and number of visits per patient as predictors. Model discrimination was summarised by ROC AUC of predicted missingness, with higher AUC indicating stronger predictability from observed covariates. Model significance was assessed using the likelihood-ratio test (Supplementary Table 3).

Statistical comparison of ROC AUC

To compare the best-performing experiment with competing experiments, we analysed fold-wise ROC AUC values across the 25 matched cross-validation folds. Because performance estimates were paired by fold, comparisons were based on paired differences in ROC AUC. Normality of these paired differences was assessed using the Shapiro–Wilk test. If the normality assumption was met, a paired t-test was applied. Otherwise, a Wilcoxon signed-rank test was used (Supplementary Table 4, Supplementary Table 11).

Cox proportional hazards model

We fitted a Cox proportional hazards model (Python packages: lifelines, scikit-survival) to estimate hazard ratios for CoA-I using the 20 most important features identified in the SHAP analysis (Figure 4a). CoA-I was defined as the event outcome, and observation time after the index visit was used as the time-to-event variable. All included predictors were numeric and standardised prior to model fitting. Hazard ratios and corresponding 95% confidence intervals were obtained from the fitted model.

Web calculator

The calculator is provided solely as a research interface for reproducibility, hypothesis generation, and external validation of the machine-learning models described in the accompanying publication. The calculator processes only structured, non-identifying clinical information; no real patient identifiers or protected health information (PHI) are stored. Gradient-like information or model internals are not linked to any individual and are used solely for technical operation and reproducibility. The scenario is treated as hypothetical and anonymized from the perspective of the application operator. It does not provide medical advice and must not be used as the sole basis for diagnosis, treatment selection, or

any other clinical decision. The interface does not provide free-text fields or fields for direct patient identifiers. Only structured, non-identifying clinical information can be entered. Clinical variables entered into the calculator are not retained by the application for model training, monitoring, or reuse. Only the deployed application and model weights are hosted. Technical access data that may be required for secure website operation, such as IP address, timestamp, and request metadata, may be processed in routine server or security logs in accordance with the institutional Privacy Notice. The calculator is not offered as a CE-marked medical device for clinical use. Its regulatory status depends on its actual intended purpose and deployment context

Reduced models employed for the web calculator

To preserve usability in routine follow-up settings, the calculator was designed to operate with a limited mandatory core set of variables, although output reliability is expected to improve as more complete phenotyping becomes available. From the 41 included features used for model development, 14 highly correlated and/or redundant features with limited additional explanatory value were removed, unless they were considered clinically relevant (Supplementary Figure 5). The web tool includes these 27 features and 3 additional variables required only for guideline-based recommendations (diameter at stenosis site, diameter of descending aorta, mean pressure drop across coarctation).

Within the web tool, only features marked with a red asterisk are mandatory user inputs: age, height, weight, resting heart rate, hypertension status, pressure gradient between upper and lower extremities, and the maximum and mean pressure drop across the coarctation on echocardiography. These variables were considered the most important either for model prediction or for guideline-based recommendations and were therefore required to ensure the minimum utility of the tool.

If non-mandatory inputs are missing, the calculator still runs using the same model-specific missing-data strategy as in model development, i.e. median/average-based imputation where required or native handling of missing values where supported.

The tool comprises two sets of prediction models: two models estimating the probability of treatment at the index visit, namely non-invasive treatment (active surveillance or medication only) and invasive intervention (balloon angioplasty, stenting, or surgery), and models predicting three post-visit outcomes over time: invasive treatment for coarctation (CoA-I), surgical treatment for coarctation (CoA-S), and arterial hypertension. These longitudinal risks are displayed as interactive plots in the web application (Supplementary Figure 4). Time-dependent probabilities were generated by varying the observation time after the index visit from 1 to 8 years.

In addition to model-based risk estimates, the tool provides guideline-directed recommendations for invasive evaluation and potential treatment based on the 2022 ACC/AHA Guideline for the Diagnosis and Management of Aortic Disease and 2024 ESC Guidelines for the management of peripheral arterial and aortic diseases (1, 2).

Hyperparameter tuning

Hyperparameters were optimised separately for each classifier using Bayesian optimisation implemented via BayesSearchCV from scikit-optimize using 5-fold stratified cross-validation to preserve class proportions across folds. For each classifier, a model-specific hyperparameter search space was defined with individual parameter boundaries (Supplementary Table 6). The optimisation was performed for up to 300 iterations per classifier, and the Matthews correlation coefficient was used as the optimisation objective. The best-performing hyperparameter configuration was refit on the full training set and used for subsequent evaluation.

Treatment effects assessment with propensity score analysis

No imputation was performed for the treatment-effects analyses. The matched sensitivity analyses were fitted as separate 1:1 comparisons for medication, balloon angioplasty, and stenting after restriction to the respective treatment category and the reference group (no treatment), using the same covariates. Due to the small number of surgical index visits, surgery was not included in the matched sensitivity analyses. CoA-S, as a subset of CoA-I, was treated as a secondary outcome and was not part of the main treatment-effects analyses. Treatment-effect estimates are reported as absolute differences in endpoint probability with 95% confidence intervals (CI).

Supplementary Table 7A reports the endpoint-specific treatment-effect estimates for CoA-I, and Supplementary Table 7B reports the corresponding estimates for future hypertension. The propensity score matching (PSM) analyses were restricted to medication (n=143), balloon angioplasty (n=149), and stenting (n=170). Surgery was not matched separately because only six surgical visits were available. Supplementary Table 8A reports the multinomial treatment-assignment model shared by the inverse-probability-weighted (IPW) analyses of CoA-I and future hypertension. Supplementary Table 8B reports the binary propensity-score models shared by the PSM analyses of CoA-I and future hypertension. Supplementary Table 9 reports the baseline group summaries, effective weighted group sizes, and post-weighting balance diagnostics for the IPW analyses of both primary endpoints. Supplementary Table 10 reports the restricted baseline group summaries, effective matched group sizes, and post-matching balance diagnostics for the PSM analyses of both primary endpoints.

References

1 Isselbacher EM, Preventza O, Hamilton Black J, Augoustides JG, Beck AW, Bolen MA, et al. 2022 ACC/AHA Guideline for the Diagnosis and Management of Aortic Disease: A Report of the American Heart Association/American College of Cardiology Joint Committee on Clinical Practice Guidelines. Circulation. 2022;146(24):e334-e482.

2 Baumgartner H, De Backer J, Babu-Narayan SV, Budts W, Chessa M, Diller GP, et al. 2020 ESC Guidelines for the management of adult congenital heart disease. Eur Heart J. 2021;42(6):563-645.

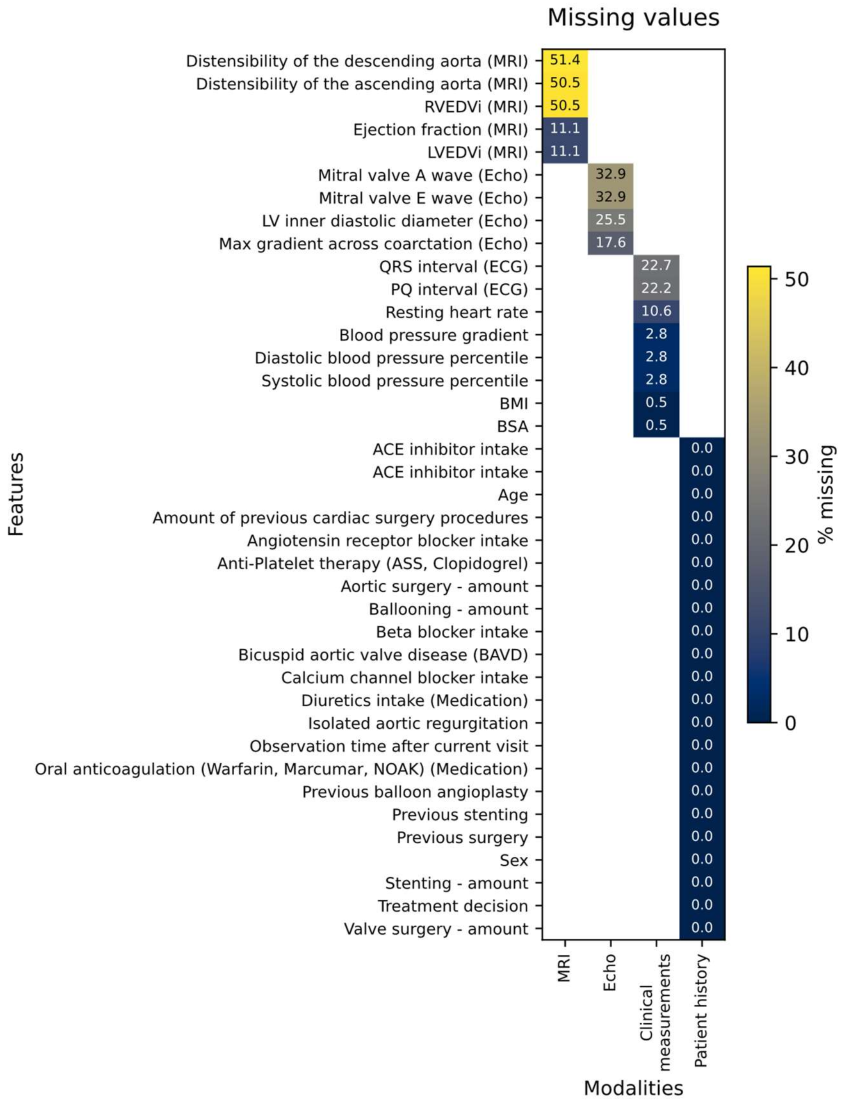

Supplementary Figure 1. Missing data across modalities

Heatmap showing the percentage of missing values for each feature across MRI, echocardiography, clinical measurements, and patient history for all visits (n = 218). Cell values indicate the proportion of missing entries (%).

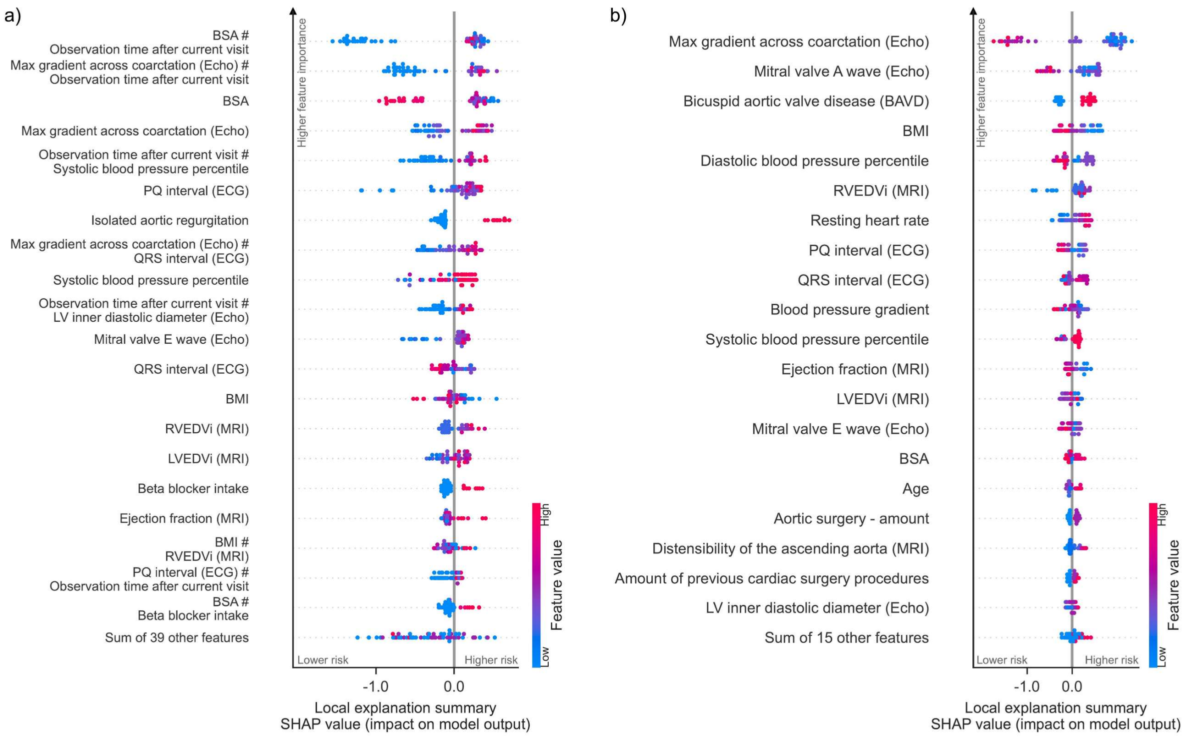

Supplementary Figure 2: Feature importance for outcome and treatment prediction based on SHAP analysis

Top 20 features ranked by mean absolute SHAP values, indicating their global importance in CoA-I with feature interactions (a) and non-invasive treatment at the index visit (b).

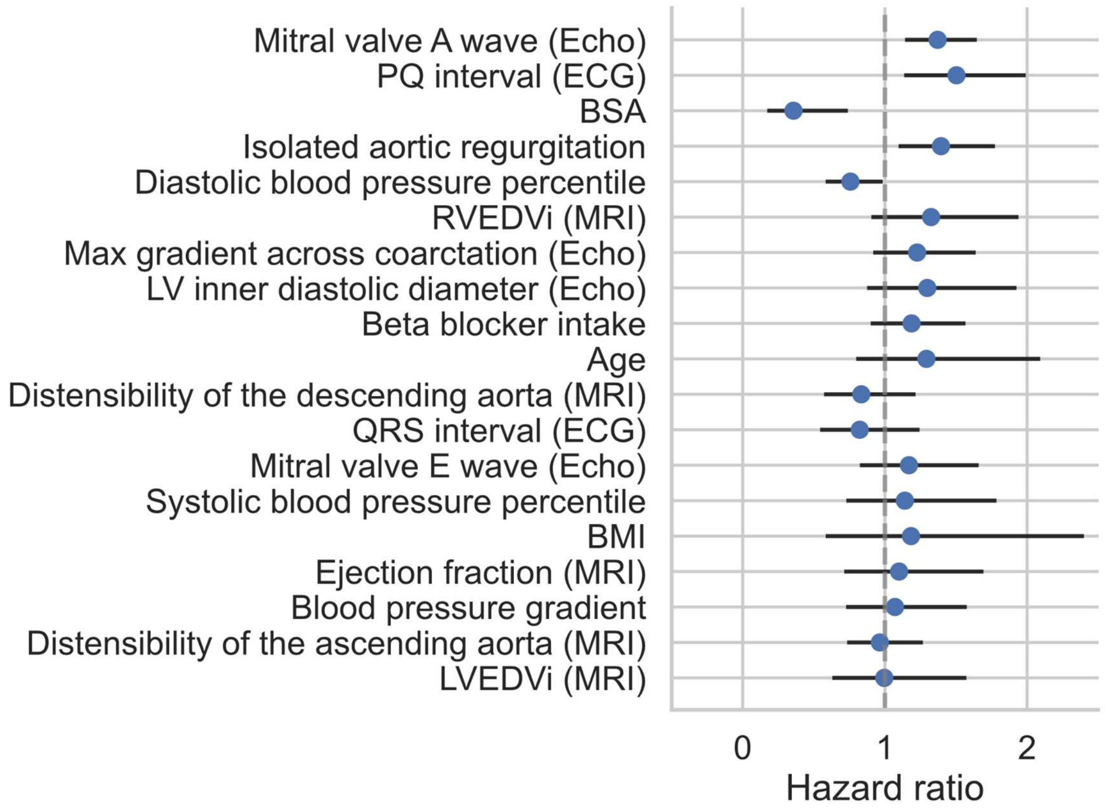

Supplementary Figure 3. Hazard ratios for CoA-I

Hazard ratios from a Cox proportional hazards model including the 20 most important features identified by the SHAP analysis for prediction of the binary CoA-I outcome (Figure 4a). Points indicate hazard ratios, and horizontal lines represent 95% confidence intervals. Mitral valve A wave (HR 1.37, 95% CI 1.14–1.65; p=0.0007), PQ interval (HR 1.50, 95% CI 1.14–1.99; p=0.0044), isolated aortic regurgitation (HR 1.39, 95% CI 1.10–1.77; p=0.0068), body surface area (HR 0.36, 95% CI 0.17–0.74; p=0.0056), and diastolic blood pressure percentile (HR 0.76, 95% CI 0.58–0.98; p=0.0375) were significantly associated with hazard of CoA-I

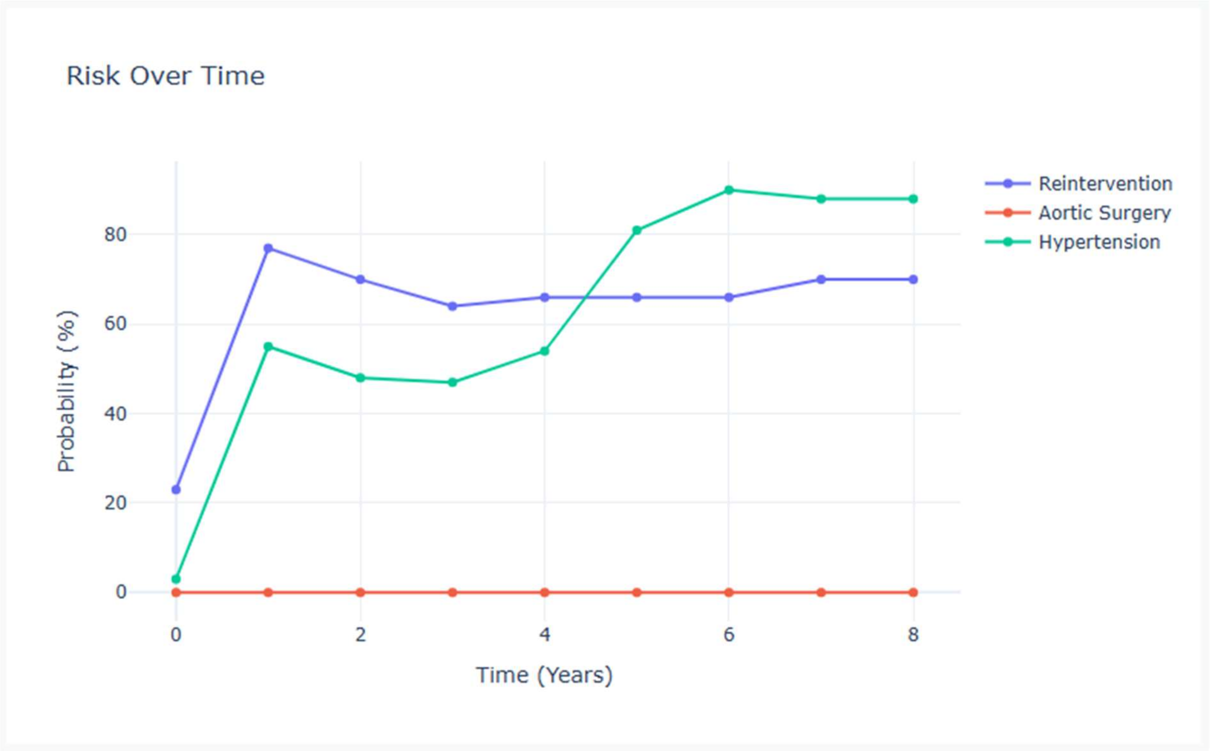

Supplementary Figure 4: Web calculator interactive plot

A screenshot of the web calculator interface, which is accessible online at: https://versnjaj.pythonanywhere.com/. The calculator allows users to input clinical and imaging features to estimate probabilities of post-visit outcomes and index-visit treatment decisions based on ML models.

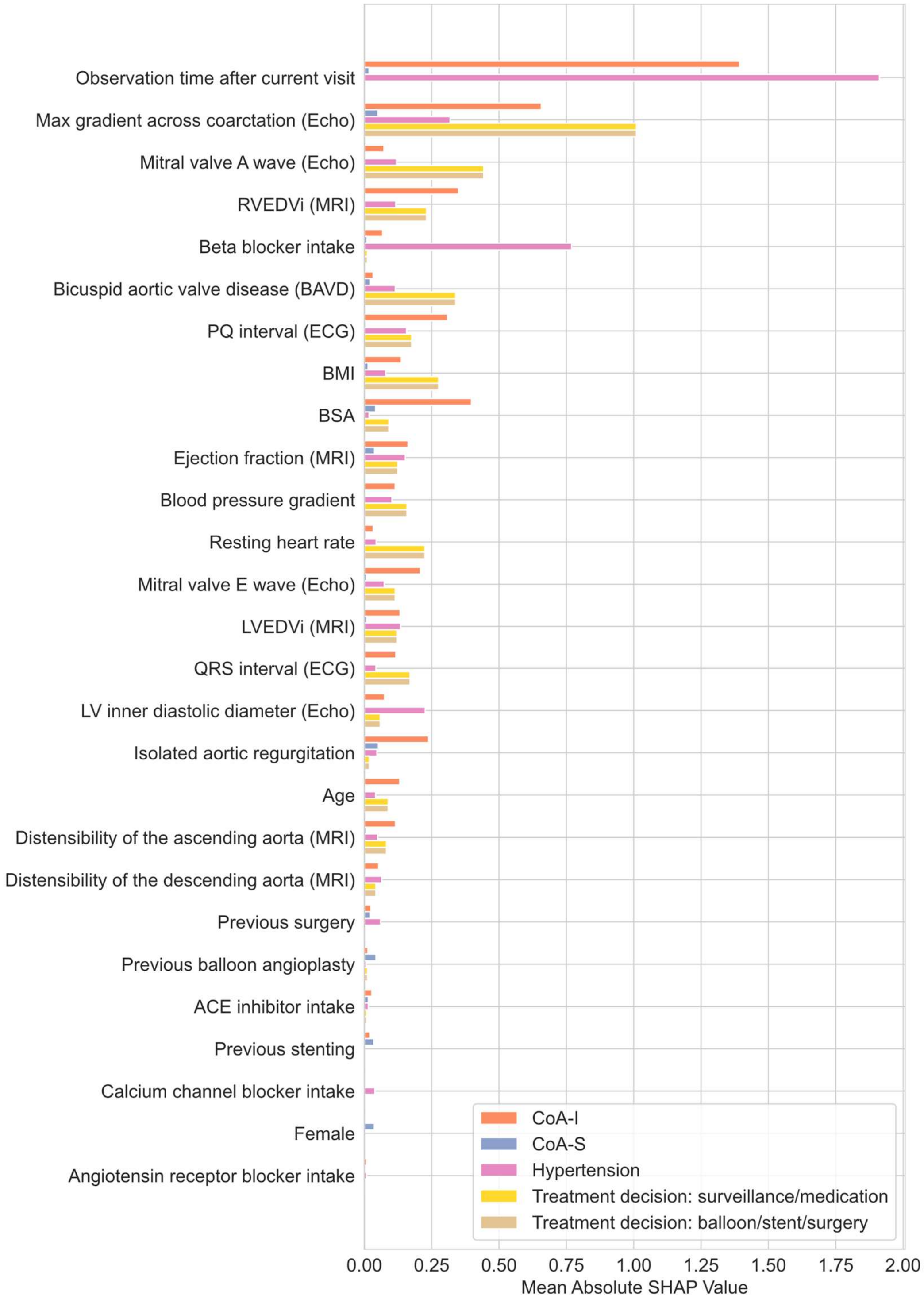

Supplementary Figure 5. Mean absolute SHAP value of features included in the reduced models

Mean absolute SHAP values of features included in the reduced models for the prediction of CoA-I, CoA-S, hypertension, and treatment decision. Higher mean absolute SHAP values indicate a greater contribution of a feature to model predictions. Observation time after the current visit was included only in the post-visit outcome prediction models.

Supplementary Table 1: TRIPOD checklist

Section/Topic	Item	Checklist Item	Page
**Title and abstract**			
Title	1	Identify the study as developing and/or validating a multivariable prediction model, the target population, and the outcome to be predicted.	1
Abstract	2	Provide a summary of objectives, study design, setting, participants, sample size, predictors, outcome, statistical analysis, results, and conclusions.	2
**Introduction**			
Background and objectives	3a	Explain the medical context (including whether diagnostic or prognostic) and rationale for developing or validating the multivariable prediction model, including references to existing models.	3
	3b	Specify the objectives, including whether the study describes the development or validation of the model or both.	3
**Methods**			
Source of data	4a	Describe the study design or source of data (e.g., randomized trial, cohort, or registry data), separately for the development and validation data sets, if applicable.	4-5
	4b	Specify the key study dates, including start of accrual; end of accrual; and, if applicable, end of follow-up.	4
Participants	5a	Specify key elements of the study setting (e.g., primary care, secondary care, general population) including number and location of centres.	4
	5b	Describe eligibility criteria for participants.	4
	5c	Give details of treatments received, if relevant.	5
Outcome	6a	Clearly define the outcome that is predicted by the prediction model, including how and when assessed.	4-5
	6b	Report any actions to blind assessment of the outcome to be predicted.	6
Predictors	7a	Clearly define all predictors used in developing or validating the multivariable prediction model, including how and when they were measured.	6-7
	7b	Report any actions to blind assessment of predictors for the outcome and other predictors.	6
Sample size	8	Explain how the study size was arrived at.	4, 5, Figure 1
Missing data	9	Describe how missing data were handled (e.g., complete-case analysis, single imputation, multiple imputation) with details of any imputation method.	6
Statistical analysis methods	10a	Describe how predictors were handled in the analyses.	6
	10b	Specify type of model, all model-building procedures (including any predictor selection), and method for internal validation.	7
	10d	Specify all measures used to assess model performance and, if relevant, to compare multiple models.	8
Risk groups	11	Provide details on how risk groups were created, if done.	-
**Results**			
Participants	13a	Describe the flow of participants through the study, including the number of participants with and without the outcome and, if applicable, a summary of the follow-up time. A diagram may be helpful.	9, Figure 1
	13b	Describe the characteristics of the participants (basic demographics, clinical features, available predictors), including the number of participants with missing data for predictors and outcome.	9, 10, Table 1
Model development	14a	Specify the number of participants and outcome events in each analysis.	9
	14b	If done, report the unadjusted association between each candidate predictor and outcome.	-
Model specification	15a	Present the full prediction model to allow predictions for individuals (i.e., all regression coefficients, and model intercept or baseline survival at a given time point).	11

	15b	Explain how to the use the prediction model.	12
Model performance	16	Report performance measures (with CIs) for the prediction model.	10, Table 2
**Discussion**			
Limitations	18	Discuss any limitations of the study (such as nonrepresentative sample, few events per predictor, missing data).	14-15
Interpretation	19b	Give an overall interpretation of the results, considering objectives, limitations, and results from similar studies, and other relevant evidence.	13-16
Implications	20	Discuss the potential clinical use of the model and implications for future research.	16
**Other information**			
Supplementary information	21	Provide information about the availability of supplementary resources, such as study protocol, Web calculator, and data sets.	Supplementary information
Funding	22	Give the source of funding and the role of the funders for the present study.	3

Supplementary Table 2: STROBE checklist

	Item No	Recommendation	Page No
**Title and abstract**	1	(*a*) Indicate the study's design with a commonly used term in the title or the abstract<br>(*b*) Provide in the abstract an informative and balanced summary of what was done and what was found	1, 2
**Introduction**			
Background/rationale	2	Explain the scientific background and rationale for the investigation being reported	3
Objectives	3	State specific objectives, including any prespecified hypotheses	3
**Methods**			
Study design	4	Present key elements of study design early in the paper	4
Setting	5	Describe the setting, locations, and relevant dates, including periods of recruitment, exposure, follow-up, and data collection	4-5
Participants	6	(*a*) Give the eligibility criteria, and the sources and methods of selection of participants. Describe methods of follow-up<br>(*b*) For matched studies, give matching criteria and number of exposed and unexposed	4
Variables	7	Clearly define all outcomes, exposures, predictors, potential confounders, and effect modifiers. Give diagnostic criteria, if applicable	4,5
Data sources/ measurement	8*	For each variable of interest, give sources of data and details of methods of assessment (measurement). Describe comparability of assessment methods if there is more than one group	4,5
Bias	9	Describe any efforts to address potential sources of bias	6,7
Study size	10	Explain how the study size was arrived at	4,5, Figure 1
Quantitative variables	11	Explain how quantitative variables were handled in the analyses. If applicable, describe which groupings were chosen and why	6,8
Statistical methods	12	(*a*) Describe all statistical methods, including those used to control for confounding<br>(*b*) Describe any methods used to examine subgroups and interactions<br>(*c*) Explain how missing data were addressed<br>(*d*) If applicable, explain how loss to follow-up was addressed<br>(*e*) Describe any sensitivity analyses	6-9, Supplementary information
**Results**			
Participants	13*	(a) Report numbers of individuals at each stage of study—eg numbers potentially eligible, examined for eligibility, confirmed eligible, included in the study, completing follow-up, and analysed<br>(b) Give reasons for non-participation at each stage<br>(c) Consider use of a flow diagram	9, Figure 1
Descriptive data	14*	(a) Give characteristics of study participants (eg demographic, clinical, social) and information on exposures and potential	9,10, Table 1

		confounders<br>(b) Indicate number of participants with missing data for each variable of interest<br>(c) Summarise follow-up time (eg, average and total amount)	
Outcome data	15*	Report numbers of outcome events or summary measures over time	9
Main results	16	(*a*) Give unadjusted estimates and, if applicable, confounder-adjusted estimates and their precision (eg, 95% confidence interval). Make clear which confounders were adjusted for and why they were included<br>(*b*) Report category boundaries when continuous variables were categorized<br>(*c*) If relevant, consider translating estimates of relative risk into absolute risk for a meaningful time period	10-12
Other analyses	17	Report other analyses done—eg analyses of subgroups and interactions, and sensitivity analyses	Supplementary information
**Discussion**			
Key results	18	Summarise key results with reference to study objectives	13
Limitations	19	Discuss limitations of the study, taking into account sources of potential bias or imprecision. Discuss both direction and magnitude of any potential bias	14-15
Interpretation	20	Give a cautious overall interpretation of results considering objectives, limitations, multiplicity of analyses, results from similar studies, and other relevant evidence	13-16
Generalisability	21	Discuss the generalisability (external validity) of the study results	15, 16
**Other information**			
Funding	22	Give the source of funding and the role of the funders for the present study and, if applicable, for the original study on which the present article is based	3

*Give information separately for exposed and unexposed groups.

Supplementary Table 3: Predictability of missingness from observed variables

For each feature with >10% missing values across visits (n=218), we modelled a binary missingness indicator (missing vs observed) using the following covariates: age, CoA-I, number of visits per patient, sex, and treatment decision. The table reports the feature-wise missing rate, the ROC AUC for predicting missingness, and the corresponding likelihood-ratio p-value testing whether the covariates jointly explain missingness.

**Feature**	**Missing rate (%)**	**Missingness ROC AUC**	**p-value**
QRS interval (ECG)	22,69	0,8	<0.001
PQ interval (ECG)	22,22	0,77	<0.001
Ejection fraction (MRI)	11,11	0,77	0,006
LVEDVi (MRI)	11,11	0,77	0,006
Mitral valve A wave (Echo)	32,87	0,75	<0.001
Mitral valve E wave (Echo)	32,87	0,75	<0.001
Max gradient across coarctation (Echo)	17,59	0,74	0,004
LV inner diastolic diameter (Echo)	25,46	0,72	0,003
Resting heart rate	10,65	0,72	0,062
Distensibility of the ascending aorta (MRI)	50,46	0,68	0,022
Distensibility of the descending aorta (MRI)	51,39	0,66	0,058
RVEDVi (MRI)	50,46	0,65	0,022

Supplementary Table 4: Sensitivity analyses in the development dataset

The best-performing classifier from Supplementary Table 11 was re-evaluated under multiple settings using 25 stratified random splits (n=159). The default model was trained with BorderlineSMOTE upsampling and median imputation. Sensitivity analyses included: (i) a reduced model using the 27 most predictive features (used for web calculator), (ii) alternative imputation strategies (mean, iterative), and (iii) class-weighted training without upsampling. Mean ROC AUC differences and corresponding p-values are reported relative to the default model. Performance metrics are presented as mean (SD), with the best performance highlighted in bold. Alternative imputation strategies did not materially improve performance; the largest mean ROC AUC improvement was 0.008 (p=0.17) for the CoA-I interaction model, and median imputation was therefore retained for the final models.

CoA-I (feature interactions)

	F1		ROC AUC				Sensitivity		Specificity		Accuracy		Precision		NPV		PR AUC	
	Train	Test	Train	Test	Mean Diff	p-value	Train	Test	Train	Test	Train	Test	Train	Test	Train	Test	Train	Test
Default	0.90 (0.02)	0.66 (0.06)	0.97 (0.01)	0.78 (0.05)	-	-	0.95 (0.02)	0.60 (0.12)	0.86 (0.02)	0.77 (0.06)	0.90 (0.02)	0.73 (0.05)	0.87 (0.02)	0.47 (0.08)	0.95 (0.02)	0.85 (0.04)	0.97 (0.01)	0.56 (0.10)
**Imputation - mean**	0.90 (0.02)	0.69 (0.06)	0.97 (0.01)	**0.79 (0.06)**	**-0.008**	0,17	0.95 (0.02)	0.65 (0.11)	0.85 (0.02)	0.77 (0.07)	0.90 (0.02)	0.74 (0.05)	0.87 (0.02)	0.50 (0.10)	0.94 (0.02)	0.87 (0.04)	0.97 (0.01)	0.58 (0.11)
Imputation - iterative	0.90 (0.02)	0.68 (0.05)	0.97 (0.01)	0.78 (0.06)	0.005	0,46	0.95 (0.02)	0.64 (0.12)	0.85 (0.02)	0.77 (0.06)	0.90 (0.02)	0.73 (0.05)	0.86 (0.02)	0.49 (0.08)	0.94 (0.02)	0.86 (0.04)	0.97 (0.01)	0.56 (0.10)
w/o upsampling	0.84 (0.02)	0.68 (0.07)	0.97 (0.01)	0.78 (0.06)	0.000	0,99	0.96 (0.02)	0.68 (0.11)	0.83 (0.02)	0.73 (0.08)	0.86 (0.02)	0.72 (0.06)	0.67 (0.03)	0.48 (0.10)	0.98 (0.01)	0.87 (0.04)	0.92 (0.02)	0.56 (0.11)

CoA-I

	F1		ROC AUC				Sensitivity		Specificity		Accuracy		Precision		NPV		PR AUC	
	Train	Test	Train	Test	Mean Diff	p-value	Train	Test	Train	Test	Train	Test	Train	Test	Train	Test	Train	Test
Default	0.89 (0.01)	0.65 (0.06)	0.96 (0.01)	0.78 (0.05)	-	-	0.94 (0.02)	0.57 (0.12)	0.84 (0.03)	0.77 (0.07)	0.89 (0.01)	0.72 (0.05)	0.85 (0.02)	0.46 (0.09)	0.94 (0.02)	0.84 (0.03)	0.96 (0.01)	0.54 (0.10)
Reduced model	0.88 (0.02)	0.67 (0.06)	0.96 (0.01)	0.78 (0.05)	-0.001	0,79	0.93 (0.02)	0.60 (0.11)	0.83 (0.03)	0.78 (0.07)	0.88 (0.02)	0.73 (0.05)	0.85 (0.02)	0.48 (0.08)	0.92 (0.02)	0.85 (0.03)	0.96 (0.01)	0.56 (0.10)
Imputation - mean	0.89 (0.02)	0.66 (0.06)	0.96 (0.01)	0.77 (0.05)	0.004	0,51	0.94 (0.02)	0.59 (0.12)	0.85 (0.02)	0.77 (0.07)	0.89 (0.02)	0.72 (0.05)	0.86 (0.02)	0.47 (0.11)	0.93 (0.02)	0.85 (0.04)	0.96 (0.01)	0.55 (0.09)
Imputation - iterative	0.89 (0.02)	0.66 (0.07)	0.96 (0.01)	0.78 (0.07)	-0.001	0,86	0.95 (0.01)	0.58 (0.15)	0.83 (0.03)	0.77 (0.07)	0.89 (0.02)	0.72 (0.06)	0.85 (0.02)	0.47 (0.11)	0.94 (0.02)	0.85 (0.05)	0.96 (0.01)	0.56 (0.09)
**w/o upsampling**	0.83 (0.02)	0.67 (0.06)	0.96 (0.01)	**0.78 (0.07)**	**-0.008**	0,24	0.96 (0.03)	0.64 (0.11)	0.81 (0.02)	0.74 (0.08)	0.85 (0.02)	0.72 (0.06)	0.65 (0.03)	0.47 (0.10)	0.98 (0.01)	0.86 (0.04)	0.90 (0.03)	0.56 (0.11)

CoA-S

	F1		ROC AUC				Sensitivity		Specificity		Accuracy		Precision		NPV		PR AUC	
	Train	Test	Train	Test	Mean Diff	p-value	Train	Test	Train	Test	Train	Test	Train	Test	Train	Test	Train	Test
Default	1.00 (0.00)	0.73 (0.12)	1.00 (0.00)	0.87 (0.09)	-	-	1.00 (0.00)	0.43 (0.23)	1.00 (0.00)	0.98 (0.02)	1.00 (0.00)	0.94 (0.03)	1.00 (0.00)	0.68 (0.32)	1.00 (0.00)	0.96 (0.02)	1.00 (0.00)	0.57 (0.18)
Reduced model	1.00 (0.00)	0.73 (0.10)	1.00 (0.00)	0.87 (0.08)	-0.004	0,56	1.00 (0.01)	0.43 (0.19)	1.00 (0.00)	0.98 (0.02)	1.00 (0.00)	0.94 (0.02)	1.00 (0.00)	0.66 (0.25)	1.00 (0.01)	0.96 (0.01)	1.00 (0.00)	0.58 (0.17)
Imputation - mean	1.00 (0.00)	0.73 (0.11)	1.00 (0.00)	0.87 (0.09)	0.001	0,81	1.00 (0.00)	0.43 (0.21)	1.00 (0.00)	0.98 (0.02)	1.00 (0.00)	0.94 (0.02)	1.00 (0.00)	0.64 (0.31)	1.00 (0.00)	0.96 (0.02)	1.00 (0.00)	0.56 (0.19)
Imputation - iterative	1.00 (0.00)	0.73 (0.11)	1.00 (0.00)	0.86 (0.09)	0.003	0,66	1.00 (0.01)	0.44 (0.19)	1.00 (0.00)	0.98 (0.02)	1.00 (0.00)	0.94 (0.03)	1.00 (0.00)	0.65 (0.32)	1.00 (0.01)	0.96 (0.01)	1.00 (0.00)	0.57 (0.19)
**w/o upsampling**	0.97 (0.02)	0.73 (0.12)	1.00 (0.00)	**0.88 (0.10)**	**-0.012**	0,17	1.00 (0.00)	0.44 (0.20)	0.99 (0.01)	0.97 (0.03)	0.99 (0.01)	0.93 (0.03)	0.90 (0.06)	0.59 (0.30)	1.00 (0.00)	0.96 (0.02)	1.00 (0.00)	0.60 (0.22)

Persistent arterial hypertension

	F1		ROC AUC				Sensitivity		Specificity		Accuracy		Precision		NPV		PR AUC	
	Train	Test	Train	Test	Mean Diff	p-value	Train	Test	Train	Test	Train	Test	Train	Test	Train	Test	Train	Test
Default	0.90 (0.02)	0.74 (0.06)	0.97 (0.01)	0.86 (0.05)	-	-	0.96 (0.02)	0.79 (0.11)	0.83 (0.04)	0.72 (0.07)	0.90 (0.02)	0.75 (0.06)	0.85 (0.03)	0.62 (0.06)	0.96 (0.02)	0.86 (0.06)	0.96 (0.01)	0.76 (0.09)
Reduced model	0.89 (0.02)	0.74 (0.05)	0.97 (0.01)	0.86 (0.04)	-0.006	0,19	0.96 (0.02)	0.81 (0.09)	0.82 (0.04)	0.71 (0.07)	0.89 (0.02)	0.75 (0.05)	0.85 (0.03)	0.62 (0.06)	0.96 (0.02)	0.87 (0.05)	0.96 (0.01)	0.77 (0.08)
Imputation - mean	0.90 (0.02)	0.74 (0.06)	0.97 (0.01)	0.86 (0.05)	-0.004	0,38	0.96 (0.01)	0.78 (0.11)	0.83 (0.03)	0.72 (0.07)	0.90 (0.02)	0.75 (0.05)	0.85 (0.02)	0.62 (0.06)	0.96 (0.02)	0.86 (0.06)	0.96 (0.01)	0.76 (0.09)
Imputation - iterative	0.90 (0.02)	0.74 (0.06)	0.97 (0.01)	0.86 (0.05)	-0.000	0,97	0.97 (0.01)	0.79 (0.09)	0.83 (0.03)	0.72 (0.07)	0.90 (0.02)	0.74 (0.06)	0.85 (0.02)	0.62 (0.07)	0.96 (0.02)	0.86 (0.05)	0.96 (0.01)	0.76 (0.09)
**w/o upsampling**	0.88 (0.02)	0.76 (0.06)	0.97 (0.01)	**0.87 (0.05)**	**-0.013**	<0.01	0.95 (0.02)	0.79 (0.09)	0.84 (0.03)	0.75 (0.07)	0.88 (0.02)	0.77 (0.06)	0.77 (0.03)	0.65 (0.08)	0.97 (0.01)	0.87 (0.05)	0.93 (0.02)	0.77 (0.09)

Active surveillance or medication only

	F1		ROC AUC				Sensitivity		Specificity		Accuracy		Precision		NPV		PR AUC	
	Train	Test	Train	Test	Mean Diff	p-value	Train	Test	Train	Test	Train	Test	Train	Test	Train	Test	Train	Test

Default	0.86 (0.01)	0.71 (0.06)	0.94 (0.01)	0.78 (0.06)	-	-	0.84 (0.02)	0.79 (0.08)	0.88 (0.02)	0.64 (0.12)	0.86 (0.01)	0.74 (0.06)	0.88 (0.02)	0.81 (0.05)	0.85 (0.02)	0.62 (0.09)	0.94 (0.01)	0.86 (0.04)
Reduced model	0.85 (0.02)	0.71 (0.06)	0.93 (0.01)	0.78 (0.06)	0.001	0,75	0.84 (0.02)	0.76 (0.10)	0.87 (0.02)	0.67 (0.13)	0.85 (0.02)	0.73 (0.06)	0.86 (0.02)	0.82 (0.05)	0.84 (0.02)	0.61 (0.09)	0.93 (0.01)	0.86 (0.05)
Imputation - mean	0.87 (0.02)	0.71 (0.06)	0.94 (0.01)	0.78 (0.06)	0.009	0,37	0.86 (0.02)	0.79 (0.07)	0.88 (0.02)	0.64 (0.12)	0.87 (0.02)	0.74 (0.05)	0.88 (0.02)	0.81 (0.05)	0.86 (0.02)	0.63 (0.08)	0.94 (0.01)	0.85 (0.04)
Imputation - iterative	0.86 (0.02)	0.71 (0.07)	0.93 (0.01)	0.76 (0.07)	0.028	<0.01	0.85 (0.02)	0.78 (0.08)	0.87 (0.03)	0.65 (0.13)	0.86 (0.02)	0.74 (0.06)	0.86 (0.02)	0.81 (0.06)	0.85 (0.02)	0.62 (0.10)	0.93 (0.01)	0.83 (0.05)
**w/o upsampling**	0.82 (0.02)	0.72 (0.06)	0.92 (0.01)	**0.79 (0.06)**	**-0.006**	0,27	0.87 (0.02)	0.82 (0.07)	0.77 (0.04)	0.61 (0.11)	0.84 (0.02)	0.75 (0.05)	0.88 (0.02)	0.80 (0.05)	0.76 (0.03)	0.65 (0.09)	0.96 (0.01)	0.86 (0.04)

Balloon angioplasty or stenting or surgery

	F1		ROC AUC				Sensitivity		Specificity		Accuracy		Precision		NPV		PR AUC	
	Train	Test	Train	Test	Mean Diff	p-value	Train	Test	Train	Test	Train	Test	Train	Test	Train	Test	Train	Test
Default	0.86 (0.02)	0.72 (0.06)	0.94 (0.01)	0.79 (0.07)	-	-	0.88 (0.03)	0.64 (0.11)	0.85 (0.01)	0.79 (0.07)	0.86 (0.02)	0.74 (0.05)	0.85 (0.01)	0.63 (0.08)	0.87 (0.03)	0.81 (0.05)	0.94 (0.01)	0.69 (0.09)
Reduced model	0.84 (0.02)	0.73 (0.05)	0.93 (0.01)	0.80 (0.07)	-0.007	0,10	0.85 (0.03)	0.70 (0.09)	0.83 (0.02)	0.77 (0.06)	0.84 (0.02)	0.75 (0.05)	0.84 (0.02)	0.62 (0.07)	0.85 (0.03)	0.83 (0.04)	0.93 (0.01)	0.69 (0.10)
Imputation - mean	0.86 (0.02)	0.72 (0.05)	0.94 (0.01)	0.78 (0.07)	0.005	0,29	0.88 (0.02)	0.66 (0.11)	0.85 (0.02)	0.78 (0.07)	0.86 (0.02)	0.74 (0.05)	0.85 (0.02)	0.63 (0.08)	0.87 (0.02)	0.82 (0.05)	0.94 (0.01)	0.69 (0.09)
Imputation - iterative	0.85 (0.02)	0.71 (0.06)	0.93 (0.01)	0.77 (0.07)	0.022	<0.001	0.86 (0.03)	0.64 (0.09)	0.85 (0.03)	0.79 (0.08)	0.85 (0.02)	0.74 (0.06)	0.85 (0.02)	0.62 (0.10)	0.86 (0.03)	0.81 (0.04)	0.93 (0.01)	0.67 (0.09)
**w/o upsampling**	0.67 (0.04)	0.59 (0.07)	0.89 (0.01)	**0.81 (0.06)**	**-0.021**	<0.001	0.33 (0.07)	0.24 (0.09)	1.00 (0.01)	0.96 (0.04)	0.77 (0.02)	0.71 (0.04)	0.99 (0.03)	0.80 (0.21)	0.74 (0.02)	0.71 (0.03)	0.85 (0.02)	0.73 (0.09)

Supplementary Table 5: Final model selection used for validation

	Model	Imputation	Class imbalance handling
**Post-visit outcome**			
CoA-I (feature interactions)	cat	median	BordelineSMOTE
CoA-I	cat	median	BordelineSMOTE
CoA-S	svc	median	BordelineSMOTE
Persistent arterial hypertension	cat	median	w/o upsampling
**Index-visit treatment decision**			
Active surveillance/medication only	xgb	median	BordelineSMOTE
Balloon angioplasty/stenting/surgery	xgb	median	w/o upsampling

Supplementary Table 6: Optimised hyperparameters per classifier

**Classifier**	**Hyperparameter**	**Search interval / values**
Logistic Regression	C	Real(0.01, 10)
	penalty	[l2]
	solver	[newton-cg, lbfgs, liblinear]
Random Forest	n_estimators	Integer(10, 200)
	max_features	[log2, sqrt]
	bootstrap	[True]
	max_depth	Integer(2, 4)
	criterion	[gini, entropy]
	ccp_alpha	Real(0.02, 0.7)
	min_samples_leaf	Integer(2, 8)
	min_samples_split	Integer(2, 15)
	max_samples	Real(0.6, 1.0)
K-Nearest Neighbors	n_neighbors	Integer(1, 11)
	leaf_size	Integer(1, 11)
	p	Integer(1, 2)
Support Vector Machine	C	Real(0.01, 10)
	gamma	Real(0.001, 0.1)
	kernel	[rbf, poly, sigmoid]
XGBoost	n_estimators	Integer(10, 200)
	learning_rate	Real(0.001, 0.3)
	max_depth	Integer(1, 6)
	subsample	Real(0.5, 0.6)
	colsample_bytree	Real(0.5, 1.0)
	colsample_bylevel	Real(0.5, 1.0)
	min_child_weight	Integer(1, 8)
	gamma	Real(0.5, 2)
	reg_alpha	Real(0.5, 2)
	reg_lambda	Real(1.0, 3.5)
CatBoost	iterations	Integer(50, 250)
	learning_rate	Real(0.01, 0.2)
	depth	Integer(1, 10)
	colsample_bylevel	Real(0.6, 1.0)
	l2_leaf_reg	Real(1, 10)
	boosting_type	[Ordered, Plain]
Neural Network	hidden_layer_sizes	Integer(5, 30)
	alpha	Real(1e-6, 0.1), log-uniform
	solver	[lbfgs, sgd, adam]
	learning_rate_init	Real(1e-5, 1e-3), log-uniform
	max_iter	Integer(100, 500)
	activation	[logistic, tanh, relu]
	learning_rate	[adaptive]

Supplementary Table 7A: Endpoint-specific treatment-effect estimates for CoA-I

Analysis	Treatment category	Analysis sample	Estimated difference	p-value
Inverse-probability weighting	Medication	218	-17.3% (95% CI, -28.2 to -6.4)	0.002
Inverse-probability weighting	Balloon angioplasty	218	-3.0% (95% CI, -19.4 to 13.4)	0.717
Inverse-probability weighting	Stenting	218	7.2% (95% CI, -8.3 to 22.6)	0.361
Inverse-probability weighting	Surgery	218	-11.4% (95% CI, -33.7 to 11.0)	0.319
Propensity-score matching	Medication	143	-17.3% (95% CI, -31.8 to -2.8)	0.019
Propensity-score matching	Balloon angioplasty	149	-8.4% (95% CI, -25.7 to 8.9)	0.341
Propensity-score matching	Stenting	170	1.6% (95% CI, -13.5 to 16.6)	0.838

Supplementary Table 7B: Endpoint-specific treatment-effect estimates for future hypertension

Analysis	Treatment category	Analysis sample	Estimated difference	p-value
Inverse-probability weighting	Medication	218	-4.0% (95% CI, -21.9 to 13.9)	0.663
Inverse-probability weighting	Balloon angioplasty	218	2.2% (95% CI, -16.3 to 20.7)	0.816
Inverse-probability weighting	Stenting	218	17.4% (95% CI, 0.5 to 34.3)	0.044
Inverse-probability weighting	Surgery	218	42.5% (95% CI, 11.4 to 73.6)	0.007
Propensity-score matching	Medication	143	-2.8% (95% CI, -31.8 to 26.2)	0.850
Propensity-score matching	Balloon angioplasty	149	10.1% (95% CI, -12.9 to 33.0)	0.390
Propensity-score matching	Stenting	170	8.7% (95% CI, -15.2 to 32.6)	0.474

Supplementary Table 8A: Multinomial treatment-assignment model shared by the inverse-probability-weighted analyses of CoA-I and future hypertension

Treatment category	Covariate	Coefficient	SE	p-value	95% CI
Medication	Visit age	0.027	0.016	0.089	-0.004 to 0.059
Medication	Previous stenting	-1.749	0.768	0.023	-3.254 to -0.243
Medication	Previous balloon angioplasty	1.135	0.595	0.056	-0.030 to 2.301
Medication	Previous aortic surgery	-0.415	0.522	0.426	-1.438 to 0.608
Medication	Constant	-2.571	0.622	<0.001	-3.790 to -1.352
Balloon angioplasty	Visit age	-0.029	0.022	0.173	-0.072 to 0.013
Balloon angioplasty	Previous stenting	0.585	0.602	0.330	-0.594 to 1.764
Balloon angioplasty	Previous balloon angioplasty	0.966	0.672	0.150	-0.351 to 2.283
Balloon angioplasty	Previous aortic surgery	-0.627	0.510	0.219	-1.627 to 0.373
Balloon angioplasty	Constant	-1.715	0.604	0.005	-2.899 to -0.530
Stenting	Visit age	0.002	0.013	0.892	-0.023 to 0.027
Stenting	Previous stenting	-0.763	0.533	0.152	-1.807 to 0.281
Stenting	Previous balloon angioplasty	0.132	0.443	0.765	-0.735 to 1.000
Stenting	Previous aortic surgery	-0.277	0.361	0.443	-0.984 to 0.431
Stenting	Constant	-0.794	0.386	0.040	-1.549 to -0.038
Surgery	Visit age	0.011	0.028	0.707	-0.044 to 0.065
Surgery	Previous stenting	-0.759	1.414	0.591	-3.531 to 2.013
Surgery	Previous balloon angioplasty	-0.220	1.116	0.844	-2.407 to 1.968
Surgery	Previous aortic surgery	-0.106	0.855	0.901	-1.781 to 1.569
Surgery	Constant	-2.960	0.917	0.001	-4.758 to -1.162

Supplementary Table 8B: Binary propensity-score models shared by the matched sensitivity analyses of CoA-I and future hypertension

Matching model	Covariate	Coefficient	SE	p-value	95% CI
Medication	Visit age	0.029	0.017	0.076	-0.003 to 0.062
Medication	Previous stenting	-1.781	0.782	0.023	-3.312 to -0.249
Medication	Previous balloon angioplasty	1.078	0.591	0.068	-0.080 to 2.236
Medication	Previous aortic surgery	-0.407	0.528	0.441	-1.441 to 0.628
Medication	Constant	-2.590	0.619	<0.001	-3.802 to -1.377
Balloon angioplasty	Visit age	-0.038	0.022	0.089	-0.081 to 0.006
Balloon angioplasty	Previous stenting	0.724	0.616	0.240	-0.484 to 1.932
Balloon angioplasty	Previous balloon angioplasty	0.995	0.669	0.137	-0.317 to 2.307
Balloon angioplasty	Previous aortic surgery	-0.559	0.520	0.283	-1.579 to 0.461
Balloon angioplasty	Constant	-1.650	0.590	0.005	-2.806 to -0.493
Stenting	Visit age	0.003	0.013	0.842	-0.023 to 0.029
Stenting	Previous stenting	-0.767	0.536	0.152	-1.816 to 0.283
Stenting	Previous balloon angioplasty	0.129	0.443	0.770	-0.738 to 0.997
Stenting	Previous aortic surgery	-0.282	0.364	0.438	-0.996 to 0.431
Stenting	Constant	-0.809	0.390	0.038	-1.573 to -0.046

Supplementary Table 9A: Effective weighted group sizes for the inverse-probability-weighted analyses of CoA-I and future hypertension

Treatment category	Raw n	Effective weighted n
No treatment	125	43.3
Medication	18	46.1
Balloon angioplasty	24	41.3
Stenting	45	44.1
Surgery	6	43.2
Total	218	218.0

Supplementary Table 9B: Baseline group summaries for the inverse-probability-weighted analyses of CoA-I and future hypertension

Comparison	Covariate	Control raw mean	Treated raw mean
Medication	Visit age	22.632	26.389
Medication	Previous stenting	0.320	0.167
Medication	Previous balloon angioplasty	0.488	0.556
Medication	Previous aortic surgery	0.496	0.444
Balloon angioplasty	Visit age	22.632	17.750
Balloon angioplasty	Previous stenting	0.320	0.583
Balloon angioplasty	Previous balloon angioplasty	0.488	0.792
Balloon angioplasty	Previous aortic surgery	0.496	0.292
Stenting	Visit age	22.632	21.889
Stenting	Previous stenting	0.320	0.200
Stenting	Previous balloon angioplasty	0.488	0.422
Stenting	Previous aortic surgery	0.496	0.444
Surgery	Visit age	22.632	24.333
Surgery	Previous stenting	0.320	0.167
Surgery	Previous balloon angioplasty	0.488	0.333
Surgery	Previous aortic surgery	0.496	0.500

Supplementary Table 9C: Post-weighting balance diagnostics for the inverse-probability-weighted analyses of CoA-I and future hypertension

Comparison	Covariate	Raw std diff	Weighted std diff	Raw var ratio	Weighted var ratio
Medication	Visit age	0.227	-0.042	1.827	0.757
Medication	Previous stenting	-0.358	0.188	0.670	1.126
Medication	Previous balloon angioplasty	0.133	0.162	1.038	0.965
Medication	Previous aortic surgery	-0.102	0.028	1.037	1.002
Balloon angioplasty	Visit age	-0.368	-0.094	0.825	1.064
Balloon angioplasty	Previous stenting	0.542	0.088	1.156	1.069
Balloon angioplasty	Previous balloon angioplasty	0.660	0.115	0.683	0.983
Balloon angioplasty	Previous aortic surgery	-0.423	0.028	0.855	1.005
Stenting	Visit age	-0.051	-0.072	1.198	1.065

Stenting	Previous stenting	-0.274	0.023	0.746	1.019
Stenting	Previous balloon angioplasty	-0.131	0.011	0.991	0.999
Stenting	Previous aortic surgery	-0.103	-0.009	1.002	0.998
Surgery	Visit age	0.111	0.207	1.423	1.124
Surgery	Previous stenting	-0.349	-0.008	0.760	0.993
Surgery	Previous balloon angioplasty	-0.304	-0.068	1.059	0.999
Surgery	Previous aortic surgery	0.008	0.170	1.190	0.998

Supplementary Table 10A: Restricted and matched sample sizes for the propensity-score sensitivity analyses of CoA-I and future hypertension

Matching model	Restricted sample n	Control n	Treated n	Matched pseudo-sample n
Medication	143	125	18	286
Balloon angioplasty	149	125	24	298
Stenting	170	125	45	340

Supplementary Table 10B: Baseline group summaries for the propensity-score sensitivity analyses of CoA-I and future hypertension

Matching model	Covariate	Control raw mean	Treated raw mean
Medication	Visit age	22.632	26.389
Medication	Previous stenting	0.320	0.167
Medication	Previous balloon angioplasty	0.488	0.556
Medication	Previous aortic surgery	0.496	0.444
Balloon angioplasty	Visit age	22.632	17.750
Balloon angioplasty	Previous stenting	0.320	0.583
Balloon angioplasty	Previous balloon angioplasty	0.488	0.792
Balloon angioplasty	Previous aortic surgery	0.496	0.292
Stenting	Visit age	22.632	21.889
Stenting	Previous stenting	0.320	0.200
Stenting	Previous balloon angioplasty	0.488	0.422
Stenting	Previous aortic surgery	0.496	0.444

Supplementary Table 10C: Post-matching balance diagnostics for the propensity-score sensitivity analyses of CoA-I and future hypertension

Matching model	Covariate	Raw std diff	Matched std diff	Raw var ratio	Matched var ratio
Medication	Visit age	0.227	-0.134	1.827	0.668
Medication	Previous stenting	-0.358	0.090	0.670	1.071
Medication	Previous balloon angioplasty	0.133	0.182	1.038	0.975
Medication	Previous aortic surgery	-0.102	0.000	1.037	1.000
Balloon angioplasty	Visit age	-0.368	0.277	0.825	1.673
Balloon angioplasty	Previous stenting	0.542	0.259	1.156	1.081
Balloon angioplasty	Previous balloon angioplasty	0.660	0.262	0.683	0.897
Balloon angioplasty	Previous aortic surgery	-0.423	0.107	0.855	1.004
Stenting	Visit age	-0.051	-0.201	1.198	0.651
Stenting	Previous stenting	-0.274	0.000	0.746	1.000
Stenting	Previous balloon angioplasty	-0.131	0.012	0.991	1.001
Stenting	Previous aortic surgery	-0.103	-0.012	1.002	0.999

Supplementary Table 11: Performance comparison of ML classifiers in the development dataset

Comparison of seven ML classifiers trained on 25 random stratified splits within the development dataset (n=159): XGBoost (xgb), CatBoost (cat), random forest (rf), support vector classifier (svc), neural network (nn), logistic regression (lr), and K-nearest neighbour (knn) - in predicting outcomes (CoA-I, CoA-S, persistent arterial hypertension) and treatment decisions (active surveillance or medication only, balloon angioplasty or stenting). Metrics are reported as mean values, with standard deviations shown in parentheses. The same imputation method (median) and class imbalance handling (BorderlineSMOTE) was used for all classifiers.

CoA-I (feature interactions)

	F1		ROC AUC				Sensitivity		Specificity		Accuracy		Precision		NPV		PR AUC	
	Train	Test	Train	Test	Mean Diff	p-value	Train	Test	Train	Test	Train	Test	Train	Test	Train	Test	Train (prev=0.5)	Test (prev=0.25)
xgb	0.89 (0.01)	0.67 (0.06)	0.96 (0.01)	0.78 (0.05)	0.001	0.88	0.93 (0.02)	0.59 (0.10)	0.86 (0.02)	0.79 (0.07)	0.89 (0.01)	0.74 (0.05)	0.87 (0.02)	0.49 (0.09)	0.92 (0.02)	0.85 (0.03)	0.96 (0.01)	0.55 (0.09)
**cat**	0.90 (0.02)	0.66 (0.06)	0.97 (0.01)	**0.78 (0.05)**	-	-	0.95 (0.02)	0.60 (0.12)	0.86 (0.02)	0.77 (0.06)	0.90 (0.02)	0.73 (0.05)	0.87 (0.02)	0.47 (0.08)	0.95 (0.02)	0.85 (0.04)	0.97 (0.01)	0.56 (0.10)
rf	0.79 (0.02)	0.65 (0.07)	0.90 (0.01)	0.78 (0.06)	0.007	0.33	0.94 (0.02)	0.81 (0.11)	0.64 (0.05)	0.63 (0.10)	0.79 (0.02)	0.68 (0.07)	0.73 (0.03)	0.44 (0.07)	0.92 (0.03)	0.91 (0.05)	0.89 (0.02)	0.54 (0.11)
svc	0.99 (0.00)	0.63 (0.07)	1.00 (0.00)	0.72 (0.07)	0.066	<0.001	1.00 (0.00)	0.42 (0.13)	0.99 (0.01)	0.84 (0.07)	0.99 (0.00)	0.73 (0.05)	0.99 (0.01)	0.48 (0.11)	1.00 (0.00)	0.81 (0.03)	1.00 (0.00)	0.52 (0.08)
nn	1.00 (0.00)	0.63 (0.04)	1.00 (0.00)	0.71 (0.06)	0.072	<0.001	1.00 (0.00)	0.44 (0.11)	1.00 (0.00)	0.82 (0.07)	1.00 (0.00)	0.72 (0.04)	1.00 (0.00)	0.46 (0.09)	1.00 (0.00)	0.81 (0.03)	1.00 (0.00)	0.50 (0.07)
lr	0.88 (0.02)	0.62 (0.06)	0.94 (0.01)	0.70 (0.06)	0.086	<0.001	0.91 (0.03)	0.51 (0.10)	0.84 (0.03)	0.75 (0.07)	0.88 (0.02)	0.69 (0.06)	0.85 (0.02)	0.42 (0.08)	0.90 (0.03)	0.82 (0.03)	0.94 (0.02)	0.48 (0.09)
knn	1.00 (0.00)	0.65 (0.07)	1.00 (0.00)	0.66 (0.07)	0.128	<0.001	1.00 (0.00)	0.52 (0.14)	1.00 (0.00)	0.80 (0.06)	1.00 (0.00)	0.72 (0.06)	1.00 (0.00)	0.47 (0.11)	1.00 (0.00)	0.83 (0.04)	1.00 (0.00)	0.37 (0.08)

CoA-I

	F1		ROC AUC				Sensitivity		Specificity		Accuracy		Precision		NPV		PR AUC	
	Train	Test	Train	Test	Mean Diff	p-value	Train	Test	Train	Test	Train	Test	Train	Test	Train	Test	Train (prev=0.5)	Test (prev=0.25)
xgb	0.89 (0.01)	0.67 (0.06)	0.96 (0.01)	0.78 (0.05)	0.005	0.41	0.92 (0.02)	0.57 (0.14)	0.86 (0.02)	0.80 (0.07)	0.89 (0.01)	0.74 (0.05)	0.87 (0.01)	0.50 (0.11)	0.92 (0.02)	0.85 (0.04)	0.96 (0.01)	0.55 (0.10)
**cat**	0.89 (0.01)	0.65 (0.06)	0.96 (0.01)	**0.78 (0.05)**	-	-	0.94 (0.02)	0.57 (0.12)	0.84 (0.03)	0.77 (0.07)	0.89 (0.01)	0.72 (0.05)	0.85 (0.02)	0.46 (0.09)	0.94 (0.02)	0.84 (0.03)	0.96 (0.01)	0.54 (0.10)
rf	0.83 (0.03)	0.65 (0.07)	0.91 (0.02)	0.74 (0.08)	0.040	<0.001	0.88 (0.06)	0.59 (0.17)	0.78 (0.04)	0.75 (0.08)	0.83 (0.03)	0.71 (0.06)	0.80 (0.02)	0.45 (0.10)	0.87 (0.05)	0.85 (0.05)	0.90 (0.02)	0.51 (0.10)
svc	0.98 (0.01)	0.63 (0.06)	1.00 (0.00)	0.70 (0.07)	0.082	<0.001	0.99 (0.01)	0.42 (0.11)	0.97 (0.01)	0.83 (0.06)	0.98 (0.01)	0.73 (0.05)	0.97 (0.01)	0.47 (0.10)	0.99 (0.01)	0.81 (0.03)	1.00 (0.00)	0.49 (0.08)
nn	1.00 (0.00)	0.63 (0.04)	1.00 (0.00)	0.70 (0.07)	0.081	<0.001	1.00 (0.00)	0.46 (0.10)	1.00 (0.00)	0.81 (0.06)	1.00 (0.00)	0.72 (0.04)	1.00 (0.00)	0.46 (0.07)	1.00 (0.00)	0.81 (0.02)	1.00 (0.00)	0.49 (0.06)
lr	0.86 (0.03)	0.61 (0.05)	0.93 (0.02)	0.69 (0.06)	0.095	<0.001	0.90 (0.03)	0.50 (0.11)	0.82 (0.02)	0.74 (0.07)	0.86 (0.03)	0.68 (0.05)	0.83 (0.02)	0.40 (0.07)	0.89 (0.03)	0.81 (0.03)	0.92 (0.02)	0.47 (0.09)
knn	1.00 (0.00)	0.59 (0.06)	1.00 (0.00)	0.60 (0.06)	0.181	<0.001	1.00 (0.00)	0.48 (0.10)	1.00 (0.00)	0.72 (0.07)	1.00 (0.00)	0.66 (0.06)	1.00 (0.00)	0.37 (0.08)	1.00 (0.00)	0.80 (0.03)	1.00 (0.00)	0.32 (0.05)

CoA-S

	F1		ROC AUC				Sensitivity		Specificity		Accuracy		Precision		NPV		PR AUC	
	Train	Test	Train	Test	Mean Diff	p-value	Train	Test	Train	Test	Train	Test	Train	Test	Train	Test	Train (prev=0.5)	Test (prev=0.07)
xgb	0.98 (0.01)	0.68 (0.11)	1.00 (0.00)	0.85 (0.09)	0.013	0.41	0.99 (0.01)	0.45 (0.22)	0.97 (0.01)	0.94 (0.05)	0.98 (0.01)	0.91 (0.04)	0.97 (0.01)	0.43 (0.25)	0.99 (0.01)	0.96 (0.02)	1.00 (0.00)	0.54 (0.20)
cat	0.99 (0.00)	0.67 (0.12)	1.00 (0.00)	0.86 (0.10)	0.012	0.43	0.99 (0.01)	0.41 (0.24)	0.99 (0.01)	0.95 (0.04)	0.99 (0.00)	0.91 (0.04)	0.99 (0.01)	0.43 (0.29)	0.99 (0.00)	0.95 (0.02)	1.00 (0.00)	0.53 (0.23)
rf	0.90 (0.02)	0.61 (0.07)	0.97 (0.01)	0.81 (0.08)	0.061	<0.001	0.94 (0.04)	0.55 (0.22)	0.86 (0.02)	0.84 (0.08)	0.90 (0.02)	0.82 (0.07)	0.87 (0.02)	0.23 (0.09)	0.94 (0.04)	0.96 (0.02)	0.97 (0.01)	0.35 (0.14)
**svc**	1.00 (0.00)	0.73 (0.12)	1.00 (0.00)	**0.87 (0.09)**	-	-	1.00 (0.00)	0.43 (0.23)	1.00 (0.00)	0.98 (0.02)	1.00 (0.00)	0.94 (0.03)	1.00 (0.00)	0.68 (0.32)	1.00 (0.00)	0.96 (0.02)	1.00 (0.00)	0.57 (0.18)
nn	1.00 (0.00)	0.70 (0.12)	1.00 (0.00)	0.84 (0.09)	0.023	<0.001	1.00 (0.00)	0.48 (0.24)	1.00 (0.00)	0.95 (0.04)	1.00 (0.00)	0.91 (0.04)	1.00 (0.00)	0.46 (0.26)	1.00 (0.00)	0.96 (0.02)	1.00 (0.00)	0.51 (0.19)
lr	0.98 (0.01)	0.64 (0.09)	1.00 (0.00)	0.78 (0.12)	0.091	<0.001	0.99 (0.01)	0.48 (0.23)	0.97 (0.01)	0.90 (0.04)	0.98 (0.01)	0.87 (0.04)	0.97 (0.01)	0.29 (0.19)	0.99 (0.01)	0.96 (0.02)	1.00 (0.00)	0.47 (0.21)
knn	1.00 (0.00)	0.65 (0.09)	1.00 (0.00)	0.68 (0.12)	0.191	<0.001	1.00 (0.00)	0.42 (0.23)	1.00 (0.00)	0.93 (0.03)	1.00 (0.00)	0.90 (0.03)	1.00 (0.00)	0.35 (0.20)	1.00 (0.00)	0.95 (0.02)	1.00 (0.00)	0.21 (0.13)

Persistent arterial hypertension

	F1		ROC AUC				Sensitivity		Specificity		Accuracy		Precision		NPV		PR AUC	

	Train	Test	Train	Test	Mean Diff	p-value	Train	Test	Train	Test	Train	Test	Train	Test	Train	Test	Train (prev=0.5)	Test (prev=0.36)
xgb	0.89 (0.02)	0.75 (0.06)	0.96 (0.01)	0.85 (0.05)	0.007	0.21	0.94 (0.02)	0.76 (0.08)	0.83 (0.03)	0.76 (0.08)	0.89 (0.02)	0.76 (0.06)	0.85 (0.02)	0.65 (0.09)	0.94 (0.02)	0.85 (0.05)	0.96 (0.01)	0.75 (0.09)
**cat**	0.90 (0.02)	0.74 (0.06)	0.97 (0.01)	**0.86 (0.05)**	-	-	0.96 (0.02)	0.79 (0.11)	0.83 (0.04)	0.72 (0.07)	0.90 (0.02)	0.75 (0.06)	0.85 (0.03)	0.62 (0.06)	0.96 (0.02)	0.86 (0.06)	0.96 (0.01)	0.76 (0.09)
rf	0.83 (0.03)	0.71 (0.07)	0.92 (0.02)	0.82 (0.07)	0.038	0.01	0.88 (0.05)	0.73 (0.14)	0.79 (0.05)	0.72 (0.06)	0.83 (0.03)	0.72 (0.07)	0.81 (0.03)	0.60 (0.08)	0.87 (0.05)	0.83 (0.07)	0.92 (0.02)	0.73 (0.09)
svc	0.96 (0.01)	0.67 (0.06)	0.99 (0.01)	0.73 (0.05)	0.123	<0.001	0.96 (0.02)	0.57 (0.10)	0.96 (0.02)	0.77 (0.05)	0.96 (0.01)	0.70 (0.05)	0.96 (0.01)	0.59 (0.07)	0.96 (0.02)	0.76 (0.05)	0.99 (0.01)	0.63 (0.08)
nn	1.00 (0.00)	0.65 (0.05)	1.00 (0.00)	0.70 (0.06)	0.160	<0.001	1.00 (0.00)	0.55 (0.10)	1.00 (0.00)	0.74 (0.07)	1.00 (0.00)	0.67 (0.05)	1.00 (0.00)	0.56 (0.07)	1.00 (0.00)	0.74 (0.04)	1.00 (0.00)	0.60 (0.08)
lr	0.84 (0.03)	0.67 (0.07)	0.91 (0.02)	0.74 (0.07)	0.117	<0.001	0.84 (0.04)	0.65 (0.11)	0.84 (0.03)	0.71 (0.08)	0.84 (0.03)	0.69 (0.06)	0.84 (0.03)	0.57 (0.09)	0.84 (0.04)	0.78 (0.06)	0.91 (0.02)	0.66 (0.09)
knn	1.00 (0.00)	0.60 (0.06)	1.00 (0.00)	0.60 (0.05)	0.252	<0.001	1.00 (0.00)	0.39 (0.08)	1.00 (0.00)	0.82 (0.05)	1.00 (0.00)	0.66 (0.05)	1.00 (0.00)	0.55 (0.09)	1.00 (0.00)	0.70 (0.03)	1.00 (0.00)	0.44 (0.05)

Active surveillance or medication only

	F1		ROC AUC				Sensitivity		Specificity		Accuracy		Precision		NPV		PR AUC	
	Train	Test	Train	Test	Mean Diff	p-value	Train	Test	Train	Test	Train	Test	Train	Test	Train	Test	Train (prev=0.5)	Test (prev=0.65)
**xgb**	0.86 (0.02)	0.71 (0.07)	0.94 (0.01)	**0.78 (0.06)**	-	-	0.85 (0.02)	0.78 (0.08)	0.88 (0.02)	0.65 (0.12)	0.86 (0.02)	0.74 (0.06)	0.87 (0.02)	0.81 (0.05)	0.85 (0.02)	0.62 (0.10)	0.94 (0.01)	0.86 (0.04)
cat	0.86 (0.02)	0.69 (0.06)	0.94 (0.01)	0.75 (0.06)	0.030	<0.001	0.85 (0.03)	0.77 (0.09)	0.87 (0.02)	0.63 (0.12)	0.86 (0.02)	0.72 (0.06)	0.87 (0.02)	0.80 (0.05)	0.86 (0.02)	0.60 (0.09)	0.94 (0.01)	0.83 (0.04)
rf	0.80 (0.02)	0.69 (0.07)	0.88 (0.01)	0.76 (0.07)	0.020	0.01	0.81 (0.02)	0.77 (0.09)	0.79 (0.04)	0.61 (0.13)	0.80 (0.02)	0.72 (0.07)	0.79 (0.03)	0.79 (0.06)	0.81 (0.02)	0.60 (0.10)	0.88 (0.02)	0.83 (0.06)
svc	0.92 (0.01)	0.62 (0.07)	0.96 (0.01)	0.66 (0.07)	0.124	<0.001	0.92 (0.02)	0.72 (0.09)	0.92 (0.02)	0.52 (0.13)	0.92 (0.01)	0.65 (0.06)	0.92 (0.02)	0.75 (0.05)	0.92 (0.02)	0.50 (0.09)	0.96 (0.02)	0.77 (0.05)
nn	1.00 (0.00)	0.58 (0.07)	1.00 (0.00)	0.59 (0.08)	0.191	<0.001	1.00 (0.00)	0.69 (0.12)	1.00 (0.00)	0.47 (0.12)	1.00 (0.00)	0.62 (0.08)	1.00 (0.00)	0.71 (0.05)	1.00 (0.00)	0.46 (0.10)	1.00 (0.00)	0.72 (0.06)
lr	0.81 (0.03)	0.64 (0.05)	0.88 (0.02)	0.68 (0.07)	0.107	<0.001	0.80 (0.03)	0.71 (0.08)	0.82 (0.03)	0.59 (0.12)	0.81 (0.03)	0.67 (0.05)	0.81 (0.03)	0.77 (0.04)	0.80 (0.03)	0.52 (0.06)	0.88 (0.02)	0.79 (0.05)
knn	1.00 (0.00)	0.56 (0.06)	1.00 (0.00)	0.56 (0.05)	0.224	<0.001	1.00 (0.00)	0.74 (0.08)	1.00 (0.00)	0.37 (0.10)	1.00 (0.00)	0.62 (0.05)	1.00 (0.00)	0.69 (0.03)	1.00 (0.00)	0.44 (0.09)	1.00 (0.00)	0.68 (0.03)

Balloon angioplasty or stenting or surgery

	F1		ROC AUC				Sensitivity		Specificity		Accuracy		Precision		NPV		PR AUC	
	Train	Test	Train	Test	Mean Diff	p-value	Train	Test	Train	Test	Train	Test	Train	Test	Train	Test	Train (prev=0.5)	Test (prev=0.35)
**xgb**	0.86 (0.02)	0.72 (0.06)	0.94 (0.01)	**0.78 (0.07)**	**-**	-	0.87 (0.02)	0.65 (0.12)	0.84 (0.02)	0.80 (0.07)	0.86 (0.02)	0.75 (0.05)	0.85 (0.02)	0.63 (0.09)	0.87 (0.02)	0.81 (0.05)	0.94 (0.01)	0.69 (0.09)
cat	0.85 (0.02)	0.72 (0.06)	0.93 (0.01)	0.77 (0.07)	0.020	<0.001	0.86 (0.03)	0.65 (0.11)	0.85 (0.02)	0.79 (0.07)	0.85 (0.02)	0.74 (0.05)	0.85 (0.02)	0.63 (0.09)	0.86 (0.03)	0.81 (0.05)	0.93 (0.01)	0.68 (0.08)
rf	0.80 (0.03)	0.70 (0.06)	0.88 (0.02)	0.77 (0.07)	0.015	0.04	0.78 (0.05)	0.63 (0.12)	0.82 (0.02)	0.78 (0.07)	0.80 (0.03)	0.73 (0.06)	0.81 (0.02)	0.61 (0.09)	0.79 (0.04)	0.80 (0.05)	0.88 (0.02)	0.67 (0.09)
svc	0.91 (0.02)	0.61 (0.07)	0.97 (0.01)	0.66 (0.07)	0.130	<0.001	0.91 (0.02)	0.51 (0.13)	0.92 (0.02)	0.73 (0.08)	0.91 (0.02)	0.65 (0.06)	0.92 (0.02)	0.50 (0.09)	0.91 (0.02)	0.74 (0.05)	0.96 (0.02)	0.55 (0.09)
nn	1.00 (0.00)	0.58 (0.06)	1.00 (0.00)	0.59 (0.07)	0.196	<0.001	1.00 (0.00)	0.47 (0.12)	1.00 (0.00)	0.69 (0.07)	1.00 (0.00)	0.62 (0.06)	1.00 (0.00)	0.45 (0.08)	1.00 (0.00)	0.71 (0.05)	1.00 (0.00)	0.49 (0.08)
lr	0.81 (0.02)	0.63 (0.07)	0.88 (0.02)	0.67 (0.08)	0.117	<0.001	0.82 (0.03)	0.56 (0.12)	0.80 (0.02)	0.70 (0.08)	0.81 (0.02)	0.65 (0.07)	0.80 (0.02)	0.50 (0.09)	0.82 (0.03)	0.75 (0.06)	0.87 (0.02)	0.55 (0.09)
knn	1.00 (0.00)	0.55 (0.07)	1.00 (0.00)	0.55 (0.07)	0.235	<0.001	1.00 (0.00)	0.35 (0.09)	1.00 (0.00)	0.76 (0.09)	1.00 (0.00)	0.62 (0.07)	1.00 (0.00)	0.45 (0.13)	1.00 (0.00)	0.69 (0.04)	1.00 (0.00)	0.39 (0.05)

Supplementary Table 12: Performance metrics in the validation dataset after excluding visits from patients who appeared in both the development and validation datasets (n=24)

		F1	ROC AUC	Sensitivity	Specificity	Accuracy	Precision	NPV	PR AUC
**Post-visit outcome**	Model								
CoA-I (feature interactions)	cat	0.84 (0.04)	0.95 (0.02)	0.97 (0.07)	0.85 (0.04)	0.88 (0.03)	0.64 (0.06)	0.99 (0.02)	0.80 (0.11)
CoA-I	cat	0.84 (0.04)	0.94 (0.02)	0.90 (0.10)	0.87 (0.03)	0.88 (0.03)	0.65 (0.06)	0.97 (0.03)	0.79 (0.10)
CoA-S	svc	0.82 (0.02)	0.99 (0.01)	1.00 (0.00)	0.91 (0.01)	0.92 (0.01)	0.51 (0.05)	1.00 (0.00)	0.97 (0.08)
Persistent arterial hypertension	cat	0.79 (0.04)	0.94 (0.01)	0.97 (0.09)	0.86 (0.02)	0.88 (0.02)	0.50 (0.05)	1.00 (0.01)	0.61 (0.04)
**Index-visit treatment decision**									
Active surveillance/medication only	xgb	0.77 (0.04)	0.90 (0.02)	0.81 (0.05)	0.77 (0.07)	0.80 (0.04)	0.89 (0.03)	0.63 (0.06)	0.96 (0.01)
Balloon angioplasty/stenting/surgery	xgb	0.77 (0.04)	0.90 (0.02)	0.77 (0.07)	0.81 (0.05)	0.80 (0.04)	0.63 (0.06)	0.89 (0.03)	0.83 (0.03)

Supplementary Table 13: Performance metrics in the validation dataset including only visits from patients who appeared in both the development and validation datasets (n=38)

		F1	ROC AUC	Sensitivity	Specificity	Accuracy	Precision	NPV	PR AUC
**Post-visit outcome**	Model								
CoA-I (feature interactions)	cat	0.67 (0.03)	0.84 (0.02)	0.79 (0.06)	0.68 (0.03)	0.71 (0.03)	0.42 (0.03)	0.92 (0.02)	0.60 (0.07)
CoA-I	cat	0.69 (0.03)	0.83 (0.02)	0.78 (0.05)	0.72 (0.05)	0.73 (0.04)	0.45 (0.04)	0.92 (0.02)	0.59 (0.05)
CoA-S	svc	0.72 (0.02)	0.76 (0.03)	0.50 (0.00)	0.96 (0.01)	0.94 (0.01)	0.47 (0.07)	0.97 (0.00)	0.32 (0.05)
Persistent arterial hypertension	cat	0.66 (0.03)	0.76 (0.01)	0.76 (0.09)	0.67 (0.03)	0.69 (0.03)	0.44 (0.04)	0.89 (0.04)	0.44 (0.01)
**Index-visit treatment decision**									
Active surveillance/medication only	xgb	0.79 (0.03)	0.84 (0.02)	0.94 (0.03)	0.63 (0.06)	0.81 (0.03)	0.79 (0.03)	0.87 (0.05)	0.85 (0.02)
Balloon angioplasty/stenting/surgery	xgb	0.79 (0.03)	0.84 (0.02)	0.63 (0.06)	0.94 (0.03)	0.81 (0.03)	0.87 (0.05)	0.79 (0.03)	0.81 (0.03)
